# Biological Signal Processing with a Genetic Toggle Switch

**DOI:** 10.1371/journal.pone.0068345

**Published:** 2013-07-16

**Authors:** Patrick Hillenbrand, Georg Fritz, Ulrich Gerland

**Affiliations:** 1 Arnold Sommerfeld Center for Theoretical Physics and Center for NanoScience, Ludwig-Maximilians-Universität München, München, Germany; 2 Department of Biology I, Synthetic Microbiology, Ludwig-Maximilians-Universität München, Planegg-Martinsried, Germany; Center for Genomic Regulation, Spain

## Abstract

Complex gene regulation requires responses that depend not only on the current levels of input signals but also on signals received in the past. In digital electronics, logic circuits with this property are referred to as sequential logic, in contrast to the simpler combinatorial logic without such internal memory. In molecular biology, memory is implemented in various forms such as biochemical modification of proteins or multistable gene circuits, but the design of the regulatory interface, which processes the input signals and the memory content, is often not well understood. Here, we explore design constraints for such regulatory interfaces using coarse-grained nonlinear models and stochastic simulations of detailed biochemical reaction networks. We test different designs for biological analogs of the most versatile memory element in digital electronics, the JK-latch. Our analysis shows that simple protein-protein interactions and protein-DNA binding are sufficient, in principle, to implement genetic circuits with the capabilities of a JK-latch. However, it also exposes fundamental limitations to its reliability, due to the fact that biological signal processing is asynchronous, in contrast to most digital electronics systems that feature a central clock to orchestrate the timing of all operations. We describe a seemingly natural way to improve the reliability by invoking the master-slave concept from digital electronics design. This concept could be useful to interpret the design of natural regulatory circuits, and for the design of synthetic biological systems.

## Introduction

It is notoriously difficult to decipher the gene regulatory program of even simple organisms. Despite the ongoing massive innovation in quantitative biology, the current experimental approaches typically do not characterize the relevant set of regulatory interactions in sufficient quantitative detail to directly read out the system behavior (the physiology) from the data. For instance, in the case of bacterial chemotaxis, it has taken many years and iterations of careful experimentation and hypothesis-guided modeling to make it one of the best characterized regulatory circuits today, despite a very modest number of components involved [1]. Yet, important new insights that change our understanding of its physiology are still being obtained [2]. The conundrum is that guidance by theoretical hypotheses about how a regulatory system functions is essential to understand the system, while we already need some understanding of the design principles of biological signal processing to generate such hypotheses. Insight into these design principles may be gained from the synthetic biology approach of combining biomolecular parts to obtain various functions [3], [4]. The salient question then is which types of signal processing functions are relevant for biological organisms?

**Figure 1:**
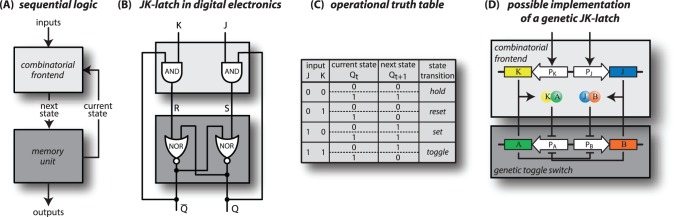
Transfer of signal processing concepts from digital electronics to molecular biology. (**A**) Schematic of a general sequential logic circuit, consisting of a combinatorial-front end and a memory unit. The front-end combines external inputs as well as the current memory state to determine the next state of the memory unit. Here, the state of the memory unit directly serves as the output of the circuit. (**B**) Wiring diagram of the JK-latch known from digital electronics, where the memory unit is typically implemented by a Set-Reset latch (composed of two cross-coupled NOR gates; *dark grey box*) and the combinatorial front-end consists of two AND gates, each of which feeds to one input of the SR-latch and integrates both the primary inputs 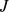 and 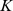 of the circuit, as well as the current state 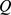 and its complement 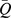 of the SR-latch (*light grey box*). (**C**) The operational truth table defines a mapping between the input signals 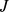 and 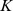 and the operation to be performed on the internal state. Each operation corresponds to a ‘state transition’ from the current state 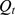 of the circuit to the follow-up state 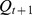. (**D**) Possible realization of a genetic JK-latch, in which the AND gates are implemented by heterodimerization between the input proteins 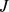 and 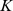 and the proteins of the memory element 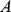 and 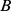. The memory element (SR-latch) is translated into a genetic toggle switch, which can be set to ON (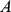 high, 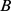 low) and reset to OFF (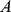 low, 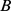 high) by additional repressor sites, binding the heterodimers 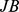 and 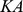, respectively.

It is clear that combinatorial logic is widely used in biological systems to integrate various signals into a single regulatory effect on a gene, effectively implementing boolean operations such as ‘AND’ or ‘NOR’, which are also fundamental signal processing operations in digital electronics [5]–[7]. Various implementations of combinatorial logics have been constructed [8]–[12], making the full variety of combinatorial logic functions available for synthetic biological systems and exploring the “design space” for such functions. However, the signal processing capability of any single combinatorial logic is very limited, since its output is always slaved to its inputs, such that, for instance, a transient input can never lead to a sustained response. An entirely new class of capabilities, called “sequential logic”, emerges when several combinatorial logics are linked together with feedback interactions, as is well known in digital electronics [13]. In contrast to combinatorial circuits, sequential logic functions maintain an internal state and condition their behavior on this internal state, making their output history-dependent. This is illustrated in Fig. 1 using the example of the so-called “JK-latch”, a sequential logic module that is frequently used in digital electronics applications. The function of this module is concisely summarized by its “truth table”, which lists the new level of the internal state (high/low) for each possible combination of the current internal and input levels. The updated internal level also represents the output of the module. For instance, one operation of this module is to “toggle” the level of the internal state, such that the output is the complement of whatever level was kept in memory before – clearly a history-dependent result.

Both feedback regulation and combinatorial regulation are common in biology, but it is not known which types of sequential logic functions are needed by organisms and how these functions are implemented. It is known, however, that even simple microbes can keep a memory of their environment, and even adaptively predict environmental changes in habitats with temporal correlations [14]–[17]. Cell differentiation in metazoans [18], and also in bacteria [19], is usually a multi-stage branching process where in each step a cell fate decision is based on differentiation steps earlier in the cell's lineage. Another interesting case of switching between alternate phenotypes, triggered by recurring environmental factors, is phase variation in pathogenic bacteria [20], [21]. Taken together, such examples suggest that sequential logic computations are in effect being performed by most organisms, although we do not yet know how.

Given these observations, it is interesting to ask how regulatory modules that implement elementary sequential logic functions could be implemented using known molecular mechanisms, and which types of sequential logics are appropriate in a biological context (rather than a digital electronics context). Here, we perform a theoretical analysis to shed some light on these questions. We first focus our analysis on the toggle operation described above, which represents a minimal version of genuine sequential logic behavior, with the output conditioned on the current internal state. The difficulties that arise in designing a biomolecular version of this functionality illustrate a more general issue to be encountered when transferring signal processing design concepts from digital electronics to biology: while sequential logic used in digital electronics is predominantly synchronous, with a central clock that orchestrates the timing of all operations, such central control is missing in biomolecular systems. Asynchronous sequential logic is avoided in digital electronics, because such circuits are known to often lead to so-called “race conditions” – conditions where the output depends sensitively on the unpredictable timing of inputs, such that it effectively becomes uncontrolled. Biological systems do not have a choice but to use asynchronous logic, resulting in design constraints that we explore.

For a logic circuit such as shown in Fig. 1B, with known wiring between nodes that have idealized properties, the intended operation can be inferred from the circuit diagram and summarized in a table like Fig. 1C. However, to inspect its actual functioning, its reliability, and its characteristic timescales, we have to consider a concrete design and study its dynamical output behavior given different dynamical inputs. For this study, we consider designs based on protein-DNA and protein-protein interactions that effect control over transcription. In particular, for the core memory unit of Fig. 1A, we chose a ‘genetic toggle switch’ consisting of two mutually repressing genes, the implementation of which was one of the first milestones in synthetic biology [22] and served as the basis for simple sequential logics [23], [24]. Genetic switches of this kind have later also been identified as important elements of the *Drosophila* gap gene network [25]. Note that for these switches the term “toggle” only refers to the capability to be set to either one of two gene expression states, not to the toggle operation, which requires the combinatorial regulation and feedback shown in Fig. 1. Of course, the memory required for any sequential logic has many other possible realizations. For instance, other switchable molecular systems include auto-phosphorylating kinases [26], [27], bistable nucleosome modifications [28], and invertible promoter switching expression of two alternate genes [29]–[32]. Since our focus is not on the memory itself, but on the interface to the memory and the feedback from it back to the regulatory “front-end”, the qualitative results of our study are more general and do not only pertain to the genetic toggle switch. Our main qualitative results are that (i) molecular sources for time delays must be introduced into the system in a certain way, such as to enable a successful toggle operation, (ii) even with these time delays, a toggle signal triggers oscillatory behavior and therefore a correct response critically depends on the duration of the applied signal, and (iii) this sensitive dependency can be resolved by interlocking positive and negative feedback loops to obtain a biological version of the so-called master-slave latch. This “master-slave circuit” obtains a reliable response to a toggle signal in a seemingly natural way, which could reflect a more general strategy in which biological systems can deal with the timing issue inherent to asynchronous signal processing systems.

## Results and Discussion

The basic building blocks of sequential logic design are called ‘latches’. They can take on two distinct states (ON/OFF or high/low) and hence store one bit of information. The output of a latch corresponds to the value of the internal state, and its operation is controled by two external inputs. The input signals are processed by a combinatorial front-end to manipulate the internal state. A latch can respond in four possible ways to input signals: it can *hold* its current state, it can be *set* to the ON state or *reset* to the OFF state, or it can *toggle* its state. The original genetic toggle switch of Gardner *et al.* [22] can perform the hold, set, and reset operations. Its front-end is particularly simple, with one input responsible for set and the other for reset, while it holds its state in the absence of inputs. This functionality corresponds to that of the Set-Reset (SR) latch, the elementary memory unit of digital electronics [13], see Fig. 1A and B. Subsequent work interfaced several biological ‘sensor modules’ to the inputs of the genetic toggle switch such that it could respond to DNA damage or local cell density (quorum sensing) [23]. More recently, a different genetic implementation of an SR-latch was reported, designed such that its switching and memory properties were more readily tunable [33]. For our study, we choose the original design of Gardner *et al.* [22] as memory unit (Fig. 1D), since our focus is on general questions of biological signal processing with such a memory unit, not so much on practical concerns for synthetic biology implementations.

### Versatile signal processing with simple protein-DNA and protein-protein interactions

A previous theoretical analysis [24] has shown that simple combinations of protein-DNA and protein-protein interactions suffice to turn a genetic toggle switch into a conditional memory unit, which memorizes a signal only when given a read “command”, akin to a Data latch in digital electronics. Here, we explore whether the same set of interactions also enable the design of a toggle command, which is intrinsically more complex, since it makes the output dependent on the memorized signal. Remarkably, a circuit capable of such a toggle operation has recently been demonstrated experimentally in a design termed ‘push-on push-off’ switch [34] (corresponding to the ‘T-latch’ of digital electronics). The circuit also uses a genetic toggle switch as memory unit, and employs UV irradiation-induced degradation of several transcription factors (via RecA). The irradiation triggers the toggle operation but is also a severe stress that kills most cells. Being a first realization of the toggle functionality, the study was focused on providing a proof of principle and not aimed at characterizing design constraints or understanding factors that affect the reliability of its operation. For instance, the intrinsically asymmetric circuit led to nearly perfect transition from the OFF to the ON state, while the success rate of the reverse transition was only about 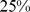. Notably, another recent experimental work used DNA reaction networks to implement the function of a T-latch *in vitro* [35]. Our theoretical study is complementary to that of Lou *et al.* [34], in that it seeks to understand the design elements needed for functional reliability.

Instead of a T-latch, we consider a more versatile sequential logic module, which is the simplest latch that can perform all four operations (hold, set, reset, toggle), controlled by just two input signals denoted J and K. A gene regulatory circuit with such a ‘JK-latch’ functionality has previously been obtained computationally using an evolutionary algorithm that selects for prescribed signal processing functions [36]. That study provided a valuable illustration of the *in silico* evolution approach, but was also not aimed at characterizing design constraints. Here, we design such circuits in a step-by-step process and test their function using coarse-grained nonlinear differential equation models as well as stochastic simulations of detailed biochemical reaction networks, allowing us to identify design constraints in the process.

Our starting point is the core memory unit of Fig. 1A, which corresponds to the SR-latch in Fig. 1B and the genetic toggle switch in Fig. 1D. The SR-latch is set to the ON-state (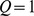) by the input 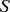 and reset to the OFF-state 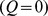 by the input 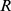. Its genetic counterpart parallels its function with two genes that mutually repress their expression through the concentrations of their gene products, the transcription factors (TFs) 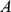 and 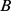. If, for instance, the expression of 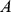 is high initially, it represses the expression of 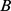 and the system remains in the (high 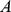, low 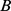)-state, i.e., it holds its state since its deterministic dynamics is bistable (however, the stochastic noise associated with gene expression will eventually lead to spontaneous memory loss) [37]. In the design depicted in Fig. 1D, the set and reset signals are mediated by two additional repressor binding sites within the promoters of genes 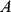 and 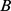, respectively, such that the concentrations of the associated repressors encode the set and reset signals. For instance, the reset signal represses the promoter of gene 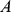 and thereby resets the switch to the (low 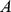, high 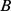)-state. Note that the contradictory input combination, where both 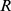 and 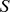 are high, is “forbidden” for the SR-latch of Fig. 1B, since this will produce a logically undefined state. For its genetic counterpart, Fig. 1D, this situation would create a random outcome, possibly with a bias towards one of the states produced by intrinsic asymmetries which are hard to avoid in the actual implementation.

The JK-latch essentially reassigns the useless contradictory input combination to the toggle operation, to become a versatile signal processing device. In the electronic circuit of Fig. 1B, this is achieved by feeding back the output 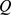 and its complement 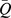 to the regulatory front-end, which combines them with the two primary inputs (denoted 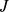 and 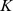). The primary inputs can still be thought of as giving set and reset commands, but due to the AND logic with the feedback signals, these commands are ignored if they would not change the internal state. For instance, if the latch is already in the ON state, a set command (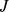 signal) is ignored. More importantly, if both commands are given simultaneously, the logic design guarantees that it will result in a change of the internal state, i.e., in a toggle operation. A gene regulatory circuit with this logic design could be obtained with the help of two auxiliary genes that perform the required AND-like signal integration on a transcriptional level. Alternatively, the signal integration could already be performed on the protein level, e.g., through heterodimerization between the TFs of the input genes and those of the toggle switch, as illustrated in Fig. 1D. This could achieve the desired characteristics without any intermediate genes, thereby avoiding the time delay and the noise associated with their expression. Heterodimerization, e.g. between 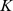 and 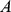, can provide an AND-type signaling logic, since only if both transcription factors are abundant, a large number of dimers form to occupy a chimeric operator, the two half-sites of which specifically bind the two monomers [38].

Would such a genetic analogue of a JK-latch indeed be able to perform the toggle operation? The intended working principle is as follows: Assume the genetic toggle switch to be in its ON-state (high 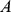, low 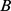) before the inputs 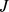 and 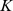 are simultaneously induced. The accumulation of 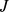 and 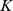 primarily leads to the formation of 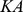 heterodimers, since 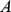 is abundant while 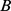 is too low to form 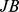. Once there are enough 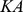 heterodimers to effectively repress the expression of 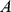, the circuit should switch to its OFF state and thereby complete the toggle operation. However, given that many nonlinear and stochastic processes interplay to determine the circuit behavior, there are potentially many ways in which it can fail. Consequently, a quantitative analysis of the circuit dynamics is required to test and understand its functioning.

**Figure 2:**
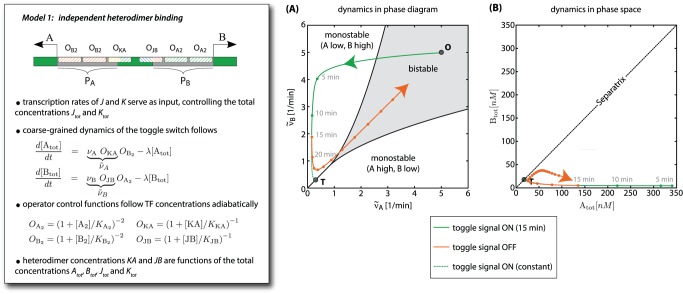
Simple JK-latch fails to perform toggle operation. (**Box**) Model 1 of the genetic JK-latch, in which heterodimers 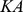 and 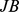 bind independently to operator sites in the promoter regions of genes 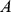 and 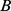, respectively (*cartoon*). In a reduced deterministic description only the total protein concentrations of the toggle switch 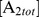 and 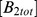 are kept as dynamic variables. In doing so, we assume that processes involving protein-DNA and protein-protein interactions adiabatically follow the current total protein concentrations, which are then described by a regulated synthesis with maximal rates 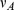 and 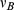 and linear degradation with rate 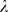. The regulatory control is described by the operator control functions 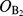, 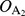, 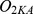 and 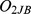 [6], [7], representing the probability to find the corresponding repressor binding site in an unbound form. To achieve the cooperativity required for the bistability of the toggle switch [39], we assume two binding sites for each of the repressor dimers 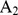 and 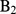. In addition, to close the rate equations for 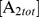 and 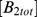 the dimer concentrations must be expressed as functions of 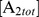, 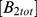, 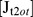 and 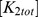, see Section 1 in File S1. (**A**) Phase diagram of the toggle switch as a function of the effective maximal transcription rates 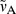 and 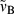. Here, the parameters are chosen such that the system is in the bistable regime in the absence of input signals (*point* **O**) and the circuit is set to the ON state initially. The curves indicate dynamic changes of 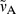 and 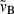, incurred by applying the toggle signal (simultaneous expression of both input genes 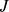 and 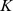) for 15 min (*green solid curve*) and then releasing it (*red curve*) or by applying it continuously (*green dotted curve*). (**B**) The same trajectories in the (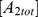,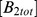)-plane. In (**A**) and (**B**) the system quickly approaches the stable fixed point **T**, representing a low A /low B state, such that a release of the toggle signal will lead to an unpredictable outcome dominated by molecular noise.

Towards this end, we first established a detailed model that explicitly accounts for the dynamics of all involved mRNA and protein species, as well as all protein-protein and protein-DNA interactions; see Table S1 in File S1 for details. Simulations of this model using Gillespie's stochastic simulation algorithm serve us as a reference (see below). However, the general behavior of the circuit is best understood within a reduced deterministic description, where only the total protein concentrations 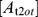 and 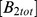 are kept as dynamic variables, as shown in the ‘Model 1’ box of Fig. 2 and detailed in Section 1 in File S1. This description assumes a separation of timescales, with protein expression and degradation as the slowest processes and protein-protein and protein-DNA interactions in quasi-equilibrium, which adiabatically follows changes in the total protein concentrations. The total concentrations of the input signal proteins are incorporated as external control variables.

Within this framework, the functioning of the circuit can be visualized as trajectories in the 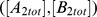-plane, see Fig. 2B, and understood by considering the two-dimensional phase diagram spanned by the effective rates of protein synthesis, 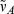 and 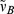, for the genes 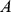 and 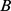, respectively, see Fig. 2A. The effective rates 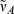 and 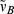 constitute the interface between the regulatory front-end and the internal memory, since they are the key variables through which the input signals control the behavior of the toggle switch: They comprise the intrinsic expression rates 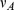 and 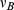 of the bare switch, i.e., the switch without the regulatory front-end, and the effect of the heterodimeric repressors, which reduce these rates. The well-known behavior of the bare switch is characterized by the simple phase diagram shown in Fig. 2A: The switch is bistable only if 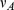 and 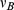 are similar and both are sufficiently strong; otherwise it is monostable, either always ON or always OFF [22], [39]. When the toggle switch is equipped with the regulatory front-end, 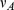 and 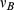 are replaced by the effective activities which are regulated by the input signals 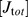 and 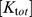. Hence, variation in the concentrations of the input signals effectively changes the intrinsic expression rates, and can thus be interpreted as “motion” in the phase diagram of the bare toggle switch.

**Figure 3:**
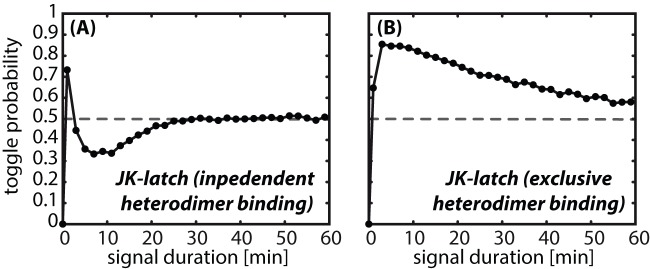
Toggle probability of the genetic JK-latch. Probability to switch in the correct follow-up state upon a toggle signal of defined duration of a J-K latch (**A**) without and (**B**) with overlapping heterodimer binding sites. Each data point is estimated by testing the final state of 5000 simulation runs of the respective full stochastic model.

With the help of the reduced deterministic description of Fig. 2 it is easy to see that the circuit must fail, and it also helps to clarify why. As shown by the colored trajectories, a toggle signal (simultaneous expression of both input genes 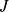 and 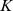) induces the system to relax to a fixed point marked as ‘T’ in Figs. 2A and B. In the phase diagram, this point lies at the boundary between the two monostable regions, where the expression level of both 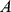 and 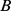 is low. Hence, after the release of the toggle signal, molecular noise will lead to an unpredictable outcome, where the switch randomly ends up either in the ON or the OFF state. A stochastic simulation of the full model for the circuit confirms this: The probability to toggle into the desired state approaches 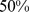 after 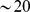 min signal duration, see Fig. 3A. Mechanistically, this failure can be explained as follows. Assuming the switch is in the (high 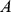, low 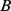)-state before receiving a toggle signal, the newly formed 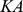 heterodimers repress gene 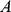, which leads to a decrease of the 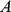 level, resulting in the derepression of gene 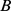. The 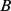 protein level rises and, since the 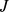 proteins from the toggle signal are still available, 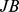 heterodimers form, again repressing gene 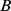 before it can reach a high expression level.

### Efficient toggle operation requires time delay

**Figure 4:**
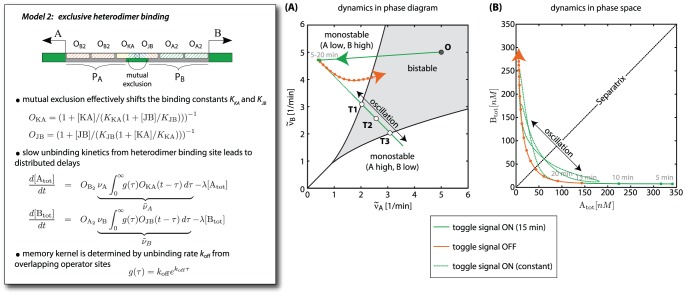
A delay mechanism is crucial for the toggle operation. (**Box**) Model 2 of the genetic JK-latch in which heterodimer binding sites overlap with their respective promoter as well as with each other, rendering heterodimer binding mutually exclusive (*cartoon*). Consequently, the operator functions 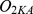 and 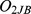 are now functions of both heterodimers: the effective binding constant of one heterodimer operator is decreased by the respective other heterodimers. Additionally, the rate of unbinding from heterodimer operators is assumed to be slow, effectively introducing a delay between the heterodimer concentration and the occupation state of their cognate operators. Since this delay is caused primarily by slow stochastic unbinding, this leads to delay-differential equation for the dynamics of 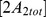 and 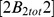 with a “memory kernel” 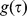. This memory kernel takes the form of an exponential distribution with rate parameter 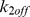, reflecting the underlying Poisson process (see Section 2 in File S1 for a detailed derivation). (**A**) In contrast to the JK-latch with independent heterodimer binding (Fig. 2), here a toggle signal drives the system rapidly to a point in the monostable regime, where it remains as long as the initial heterodimer species is bound to the overlapping operator site. Thus, if the toggle signal is taken away in time, the circuit switches its state and relaxes back to bistable regime without coming close to the complementary monostable regime. However, under a continuous toggle signal (*dotted curves*) the circuit performs oscillations through three unstable fixed points (**T1-3**). (**B**) Trajectories of the system under timed and continuous toggle signals in the (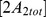,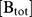)-plane.

In essence, the problem of the circuit analyzed above is that the time required to flip the switch is much longer than the timescale of the feedback regulation. Due to the rapid feedback from the state of the switch to the front-end, the switch does not reach a definite follow-up state, but instead settles into an undefined low-low state. In typical digital electronic devices this problem does not occur since the timescales of switching and feedback are given by signal propagation speed and hence are comparable. For our biomolecular circuit, the problem of mismatched timescales could be solved by introducing a mechanism that creates a time delay for the feedback. Such a delay can be achieved, for instance, by overlapping binding sites of the heterodimers 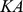 and 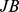, such that the transcription factors mutually exclude each other [37], [40], see Fig. 4. If the dwell time of a heterodimer, say 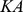, on its binding site is long enough, proteins 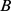 of the new state should accumulate to high levels before 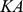 unbinds and gives way to repression of gene 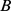 by 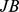.

To study this altered circuit design, we again established a reduced deterministic description, see the ‘Model 2’ box of Fig. 4, as well as a detailed stochastic model, see Table S2 in File S1. For the reduced description, we can no longer assume rapid equilibrium of protein-DNA interactions, since, in order to achieve a sufficient time delay, the rate of heterodimer unbinding, 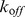, needs to be slow [41]. The dwell time 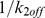 of the heterodimers on their operator then sets the timescale for the feedback, and the deterministic description is changed from two coupled ordinary differential equations (Model 1 of Fig. 2) to two coupled delay-differential equations (Model 2 of Fig. 4). More precisely, since heterodimer unbinding is a stochastic single-molecule event, the time delay 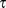 is not fixed, but has a statistical distribution, 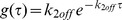, which acts as a “memory kernel” for the delay-differential equations; see Section 2 in File S1 for the derivation of the model. Intuitively, the equations in Fig. 4 describe a delayed model in the sense that the heterodimer operators effectively “see” the protein concentrations from a former point in time.

We tested the dynamics of the altered circuit within our reduced deterministic description, as well as the full stochastic model. To this end, we chose parameter values within a physiological regime (Table S3 in File S1) and performed simulations with temporally changing input signals 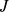 and 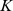. Fig. 4 displays the deterministic dynamics induced by a toggle command as a trajectory in the phase diagram of the genetic toggle switch (Fig. 4A) and as a trajectory in phase space (Fig. 4B). We consider first the case of a short toggle signal (15 min). As for the circuit of Fig. 2, the regulatory front-end rapidly moves the toggle switch into the monostable (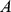 low, 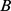 high) zone of the phase diagram (solid green arrow in Fig. 4A). However, when the signal stops, the system switches its state and relaxes back to the bistable regime without ever coming close to the complementary monostable regime (orange arrow in Fig. 4 A and B). Hence, the delay has effectively eliminated the undefined (low, low)-state that prevented a reliable toggle operation in the circuit of Fig. 2.

To test the altered circuit design with respect to toggle reliability in the presence of gene expression noise, we performed simulations of the full stochastic model. Fig. 3B shows the probability to toggle into the other state as a function of the toggle signal duration. In contrast to the circuit of Fig. 2, the toggle probability increases sharply from 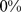 to 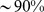 already for a toggle signal duration of 5 min. However, for longer signal durations the probability of success gradually declines, reaching 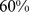 for a 60 min signal. While the precise values of the toggle success depend on the circuit parameters, see Fig. S4 in File S1, such that it could be optimized by parameter tuning, the basic feature of a declining toggle probability with increasing signal duration is parameter-invariant. This observation points towards another general problem of the circuit design, which we study next.

### Delay-induced oscillations: a genetic race condition

**Figure 5:**
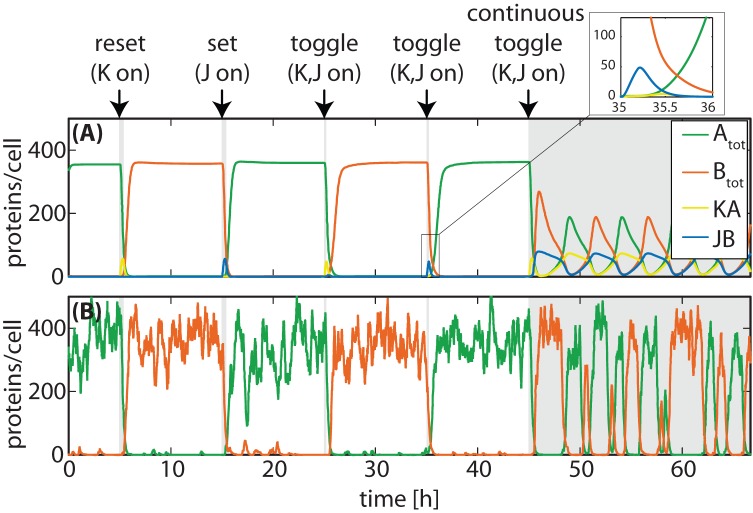
Time evolution of protein concentrations in the JK-latch upon a sequence of input signals. (**A**) Simulation by the full deterministic model, in which each biochemical species is described by one rate equation. (**B**) Stochastic simulation of the reaction system. In contrast to the deterministic model, the oscillations of the race condition occur irregularly.

To pinpoint the mechanism underlying the decreasing toggle probability, it is instructive to analyze the deterministic response of the circuit to a sustained toggle signal. Fig. 3A and B also display the dynamics for this case (dashed green lines), showing that the switch spontaneously oscillates between the ON and the OFF state. This observation is plausible, given that delays in regulatory systems with negative feedback loops are a well known mechanism for oscillators [42], [43]. A stability analysis confirms that the system undergoes a Hopf bifurcation [44], [45] beyond a critical time delay with an oscillatory period determined by the mean dwell time of the heterodimers on their operators, see Fig. S3 in File S1. Moreover, the deterministic model displays oscillatory behavior over a range of input signal strengths, see Section 4 and Fig. S2 in File S1. Fig. 5 illustrates and summarizes the behavior of the circuit by showing a time series with the input signals 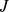 and 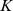 varying such that the switch is first reset, then set, followed by transient toggle signal, and finally a sustained toggle signal. Both the deterministic and the stochastic dynamics of the 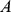, 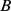 protein levels are shown. In the stochastic description, the response dynamics to the sustained toggle signal is very irregular, but still oscillatory. A more detailed characterization of the dynamics is provided in the Section 4 in File S1.

The unwanted oscillation is an example for a class of glitches referred to as “race conditions” in digital electronics, where the circuit output depends on the precise timing of input signals [13], [46]. Race conditions can occur in many sequential logic circuits: If the response of a circuit depends on its current internal state and if it changes its state during the response, then the new state feeds back to the front-end and can cause again a different follow-up state. The circuit behavior critically depends on the ratio of different timescales. Two important timescales are the time required to switch the memory state and the time delay of the feedback from the internal state back to the front-end. If, as in the circuit of Fig. 2, feedback regulation is fast compared to switching, the input from the front-end changes before the memory device can reach its new state and thus the circuit is likely to settle into an undefined state (or metastable state). If, however, the feedback is slower than the switching process, the system can reach its new state before the input from the front-end changes (as in the circuit of Fig. 3). Under this condition, a correct response can be achieved if the duration of the input signal – a third important timescale – is short enough, as seen in Fig. 5.

In digital electronics, the standard way to circumvent race conditions is to control the signal timing in the circuit with an external clock signal, which locks the state of the latches after half a period. However, such a global and deterministic clock signal is an unbiological concept, and biomolecular signal processing must rely on a different solution to this problem. Ideally, the design of genetic circuits should be insensitive to the uncertain timing of signals and lead to a reliable output independent of signal durations. Interestingly, digital electronics also offers another solution to the problem with a design concept referred to as “master-slave latch”. In the following, we study the applicability of this concept for biomolecular signal processing.

### Genetic master-slave latch

The basic idea of the master-slave latch is that the memory unit representing the state of the system – the slave circuit – is interlocked with another bistable system – the master circuit. The state of the slave circuit determines whether set or reset has to be triggered to achieve the proper response to a toggle signal. The selected signal (set or reset) is stored in the master circuit, which from thereon continuously applies this signal to the slave circuit (until the external input signals to the master circuit change). Thereby, the master circuit fixes the interpretation of the external toggle signal, preventing an oscillatory response.

**Figure 6:**
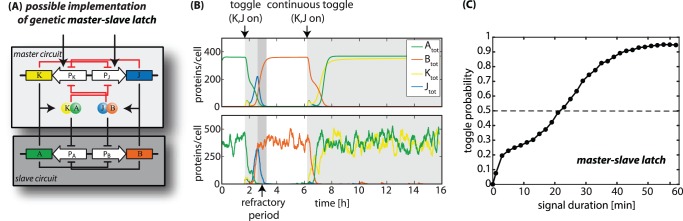
Master-slave circuit. (**A**) Possible realization of a genetic master-slave latch, which extends the genetic JK-latch in Fig. 1D by only a few interactions (*red lines*): Genes 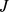 and 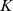 now mutually repress their expression to form a second bistable toggle switch (the master latch). Furthermore, the input genes are repressed by the heterodimers, so that the state of the master toggle switch is determined by the current state of the slave toggle switch, which stores the output state. In our model, we take the transcription rates of the bare promoters of 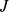 and 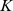 (in the absence of repressors) as external input signals. In an *in vivo* implementation such additional control could be implemented, e.g., through activator binding sites upstream of the 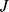 and 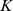 promoter regions [53]. Alternatively, one might control the transcript levels of 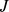 and 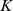 directly via small non-coding RNAs expressed from auxiliary promoters [54]. (**B**) Deterministic and stochastic time evolution of protein populations in the genetic master-slave latch upon a timed (60 min) and a continuous toggle signal. After the system has toggled once it has to undergo a refractory period, in which the signal proteins degrade before another toggle signal can be applied. (**C**) Probability of the the master-slave latch to switch in the correct follow-up state upon a toggle signal as a function of the signal duration. Each data point is estimated by testing the final state of 5000 simulation runs of the full stochastic model.

A genetic analog of this design concept is shown in Fig. 6A. Importantly, the master-slave principle removes the requirement for a specific delay mechanism, since the slave circuit only receives simple set or reset signals from the master. As a consequence, the genetic circuit can be based on the simpler design of Fig. 1D, releasing the restrictive constraints of Fig. 4A on the promoter design. Compared to the circuit of Fig. 1D there are additional regulatory interactions in Fig. 6A, which are shown in red. The inducible signal genes 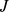 and 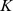 now mutually repress each other to form the master switch. Furthermore, the slave switch feeds back onto the master switch via the heterodimers 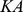 and 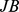. However, the action of the heterodimers in the master switch is reversed compared to the slave switch, such that 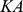 represses gene 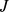 and 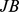 represses gene 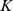.

When the master-slave circuit of Fig. 6A receives a toggle signal (simultaneous induction of genes 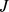 and 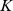, see caption), the interlocked feedback loops make the master switch sensitive to the current state of the slave switch, such that the master is forced into the 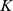 high, 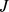 low (‘RESET’) state if 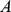 is high initially and into the 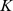 low, 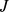 high (‘SET’) state if 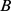 is high initially. Given that the response times of both switches are comparable, one expects that the master switch then holds the SET and RESET state irrespective of the toggle signal duration, since the proteins of the slave switch can no longer form heterodimers with the proteins of the master switch: If, for instance, the slave switch is turned OFF (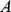 low, 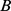 high) by the toggle signal, the master switch is simultaneously locked into the RESET state (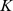 high, 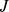 low), such that neither 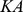 nor 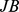 can form.

To test this circuit design, we extended the reaction model of the JK-latch by the additional regulation depicted in Fig. 6A. The deterministic response to a toggle signal and a typical stochastic trajectory are shown in Fig. 6B and Fig. S5 in File S1. The system is in the ON state initially and, after induction, both signal genes are expressed. Subsequently, the newly forming heterodimers 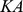 simultaneously repress transcription of genes 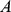 and 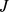, causing the slave circuit to switch into its OFF state and the master circuit to repress 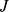. After the toggle signal is taken away, the system has a refractory period before a new toggle signal can be successfully applied. During this period, signal proteins degrade and the promoters of genes 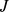 and 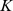 clear, such that the new state of the master circuit is no longer biased towards its former state due to repression by the homodimers 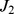 or 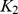.

A sustained toggle signal is next applied in Fig. 6B. Instead of falling into a race condition, the master-slave latch switches its state once and remains in this state thereafter. Thus, the master-slave latch is able perform a correct toggle response independent of the signal duration, provided the signal is not too short, see Fig. 6C. A remaining source of error is that the master switch can switch to a false state at the onset of expression of signal proteins. However, the probability for this to happen is low, leading to a maximal toggle probability of 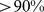 within a broad range of the system parameters – see Fig. S4 in File S1.

On a conceptual level, the master-slave principle eliminates the race condition by creating a transient redundancy between the master and the slave switch, in the sense that during the toggle command, the correct state is stored in both switches. This redundant information exists only as long as the toggle signal is applied: For instance, if the initial state is ON, then the circuit switches to RESET/OFF when a toggle signal is applied and from there to OFF when the signal is taken away eventually. This simple principle, which achieves a robust operation, could be employed to solve the inherent timing problem of sequential logic gene networks in cells that receive irregularly timed signals of variable duration. From a nonlinear dynamics point of view, the strategy consists of creating temporary stable fixed points, which are unique to the initial state and the applied signal, and which decay to the desired follow-up state after the signal is taken away. In principle, this can be done by creating a bistability in the signal itself that is regulated (switched) by the initial state. Also, the bistability does not necessarily need to be in gene expression levels – for instance, bistability in signaling phosphorelay cascades is another possible mechanism.

## Conclusions

It is clear, in principle, that gene regulatory circuits can perform logical operations involving internal memory (sequential logic), however it is not known to what extent this capability is used in natural systems. Here, we studied designs of minimal genetic circuits for sequential logic from a theoretical perspective, complementing the ongoing experimental effort in synthetic biology. We considered biological analogs of the most versatile memory element in digital electronics, the JK-latch. Our analysis suggests that combinations of simple protein-protein and protein-DNA interactions can implement JK-latch-like behavior, but such circuits would fail to function reliably in realistic biological settings. In contrast to simple combinatorial logics, sequential logic operations, in particular those that involve flipping the state of the internal memory (such as the toggle operation of the JK-latch), are very sensitive to dynamical properties of the circuit. In combinatorial circuits, a unique steady state response to an input signal is always reached, regardless of how fast individual modules within the circuit work relatively to each other. In contrast, the feedback in sequential logic circuits leads to a critical sensitivity on the ratio of timescales of key processes: Our analysis has shown that the genetic JK-latch toggles its state only if the feedback from the memorized state to the regulatory front-end has a slower timescale than the switching process of the internal state. Then, however, the JK-latch falls in an oscillatory state (‘race condition’) under a toggle signal, in which it switches back and forth between the internal states. This makes the success of the toggle transition crucially dependent on an appropriate timing of the input signals.

These issues typically do not occur in digital electronics, where the timescales are fundamentally different and a central clock usually controls the timing of signals. However, a digital electronics design concept does suggest a strategy to resolve these issues, leading to a genetic circuit that can function reliably in a biological setting. The central idea of this master-slave concept is to generate interlocked feedback loops which lock down the internal state after a toggle operation. In other words, a beginning oscillation of the internal state is stopped exactly after half a period, such that the state switches reliably, independent of the duration of the toggle signal. This design is achieved by additional feedbacks from the internal state to the regulatory front-end, and within the front-end itself.

Similar issues can occur in other biomolecular circuits that control internal states of cells in a signal-dependent manner. A natural context in which such functions are required is the differentiation of cells or regulated phenotype-switching. The master-slave concept of interlocked feedback loops is an elegant general strategy to counteract issues generated by the asynchronous and unreliable timing of biological signals. A test of this concept using the synthetic biology approach is highly desirable. We speculate that circuits involving the master-slave principle will eventually also be identified in natural regulatory circuits.

## Methods

### Models of gene regulatory circuits

All genetic circuits were modeled by first setting up a list of biochemical reactions, describing all relevant processes involved. These include transcription and translation, protein-DNA binding, dimerization and mRNA and protein degradation (see Fig. S1 and Tables S1, S2 and S4 in File S1 for an overview of the reactions in the individual circuits; all models are also accessible in SBML format as Supporting Material). Note that the maximal transcription rates of genes 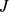 and 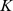 are considered as time-dependent input signals in all circuits. Transcription of each gene proceeds at this maximal rate if its cognate promoter is free to bind RNA Polymerase. Likewise, transcription of each gene is blocked if at least one repressor dimer is bound to the cognate promoter region. The resulting mRNA is translated to protein and is linearly degraded. Proteins can reversibly dimerize and bind to promoters as repressors. Protein monomers and dimers are linearly degraded with equal rates, that is, we do not assume cooperative stability [47]. The kinetic parameters for the reactions were chosen from physiologically reasonable regimes (Tables S3 and S5 in File S1). Note that all on-rates of protein-protein and protein-DNA reactions were assumed to be diffusion-limited [48] and the off-rates were chosen to attain physiological dissociation constants accordingly. Furthermore, to make switching of the circuits temporally efficient, fast active protein degradation by SsrA tags with a protein half-life of 5 min was assumed [49].

### Deterministic analysis

Assuming spatial homogeneity, the biochemical reaction equations of a circuit were translated into a set of deterministic rate equations for the average concentrations of all reactants [50], which we refer to as full deterministic model. By assuming a separation of timescales, this full deterministic model was condensed to an effective reduced model, in which only total protein concentrations are treated as dynamic variables and all dimerization and protein-DNA reactions are assumed to be in quasi-equilibrium, see Section 1 in File S1. However, for the genetic JK-latch with overlapping heterodimer operators this quasi-equilibrium assumption breaks down. In that case, the representation of only one variable per gene can be retained by incorporating the operator dynamics in form of a distributed delay into the protein concentration dynamics, leading to the delay-differential equations in the box of Fig. 4, as detailed in Section 2 in File S1.

The equations of the full deterministic model and the reduced model without delay were solved numerically using custom-built C++ code. The delay-differential equations were solved by storing a history of the system state, which was then used to numerically calculate the integrals on the right hand side of the equations. In Section 3 in File S1 an adapted linear stability analysis for delay-differential equations is presented. Additional to partial derivatives of the system, Laplace transforms of the delay kernel enter the characteristic equation [51]. This stability analysis is used to determine the stability of the system under variation of certain parameters, as described in Section 4 in File S1.

### Stochastic analysis

To account for stochastic effects the full set of biochemical reactions was simulated directly by Gillespie's stochastic simulation algorithm [52]. From 5000 repeated simulation runs the toggle probability in Figs. 4 and S4 was estimated as follows: In each simulation the system was equilibrated without input signals for 100 min, then the toggle signal (high transcription rates of 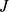 and 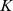) was applied for a defined period and the simulation terminated after the last input protein was decayed. If in the final configuration 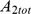 was bigger than 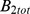 the final state was counted to be in the ON state and to be in the OFF state *vice versa*.

## Supporting Information

File S1 **Supporting text, tables and figures.** Contains detailed reaction equations and parameter choices of all discussed circuits, derivations and additional analysis of all reduced models and details on linear stability analysis of delayed differential equations.
(PDF)

File S2 **Model files for all circuits in SBML format.** Contains SBML files for the J-K latch with and without overlapping heterodimer binding sites and the master-slave latch.
(ZIP)
