# Supplementary material for: Biological Signal Processing with a Genetic Toggle Switch
Source: PLoS One. 2013 Jul 16;8(7):e68345. doi: 10.1371/journal.pone.0068345 (PMC3712956; doi:10.1371/journal.pone.0068345)
Supplement: File S1 — Supporting text, tables and figures. Contains detailed reaction equations and parameter choices of all discussed circuits, derivations and additional analysis of all reduced models and details on linear stability analysis of delayed differential equations. (PDF) [file pone.0068345.s001.pdf]

Supplementary Material to: Biological signal processing with a genetic toggle switch

Patrick Hillenbrand[1], Georg Fritz[1,2], Ulrich Gerland[1,*]

1 Arnold Sommerfeld Center for Theoretical Physics (ASC) and Center for NanoScience (CeNS), Ludwig-Maximilians-Universität München, Theresienstr. 37, 80333 München, Germany

2 Department of Biology I, Synthetic Microbiology, Ludwig-Maximilians-Universität München, Großhaderner Str. 2–4, 82152 Planegg-Martinsried, Germany

$*$ **E-mail: gerland@lmu.de**

1 Derivation of a reduced model for the JK-latch with independent heterodimer binding sites

The reactions describing the genetic JK-latch, presented in Table S1 and S2 can be classified into three different categories: protein-protein reactions (including protein degradation), protein-DNA reactions and gene expression (including transcription and degradation of mRNA and translation into proteins). A full deterministic model can be derived by setting up a rate equation for the mean concentration of each biochemical species in the reactions. A first step towards a reduced model is to assume the occupation states of the promoters (that is the protein-DNA reactions) to be in equilibrium with the current transcription factor concentrations at all times. Since all TF's act as repressors, we assume that the only promoter state contributing to mRNA production is the unoccupied one. Therefore the rate equation for mRNA of, for instance, gene A is,

$$\frac{dm_A(t)}{dt} = \nu_{m_A} P_A(t) - \lambda_m m_A(t)\,, \tag{1}$$

where ν_{m_A} is the maximal transcription rate, λ_m the mRNA degradation rate and $P_A(t)$ denotes the promoter activity function, that is, the probability of the promoter to be unoccupied and free to bind RNA polymerase at a certain point in time. Hence, assuming all protein-DNA reactions to be in equilibrium, $P_A(t)$ describes the equilibrium probability to find the promoter unoccupied as a function of current transcription factor concentration. This probability can also be calculated by thermodynamic models,

corresponding to [1,2]. Their specific form for the genetic JK-latch is defined in the boxes of Figs. 2 and 3.

As a further simplification, we assume all protein-protein reactions to rapidly reach equilibrium with the current total concentration of reacting proteins. For instance, for the concentrations of all protein species containing A and K this leads to the following set of equations[1]

$$A_{\mathrm{tot}} = A + 2A_2 + KA\,, \tag{2}$$

$$k_{A2}^{\mathrm{on}} A^2 - k_{A2}^{\mathrm{off}} A_2 - \lambda_p A_2 = 0\,, \tag{3}$$

$$k_{KA}^{\mathrm{on}} K\cdot A - k_{KA}^{\mathrm{off}} KA - \lambda_p KA = 0\,, \tag{4}$$

where we can additionally set $K = K_{\mathrm{tot}} - KA$. After solving this system for the concentrations of homo- and heterodimers all promoter activity functions in Figs. 2 and 3 of the main text can then be expressed in terms of total concentrations, i.e., $P_A(B_2, KA) = P_A(A_{\mathrm{tot}}, B_{\mathrm{tot}}, K_{\mathrm{tot}}, J_{\mathrm{tot}})$.

Altogether, we are left with two equations left to describe the total concentration of each gene product. For instance, the equations for gene product A are

$$\frac{dA_{\mathrm{tot}}(t)}{dt} = \nu_{p_A} m_A(t) - \lambda_p A_{\mathrm{tot}}(t)\,, \tag{5}$$

$$\frac{dm_A(t)}{dt} = \nu_{m_A} P_A(t) - \lambda m_A(t)\,. \tag{6}$$

As a last step, we set Eq. (6) to zero thereby assuming mRNA translation to be fast with respect to transcription (we will discuss this assumption in the next section). Solving the resulting equation for m_A and substituting it in Eq. (5) leads to

$$\frac{dA_{\mathrm{tot}}(t)}{dt} = \nu_A P_A(t) - \lambda_p A_{\mathrm{tot}}(t)\,, \tag{7}$$

with an effective maximal expression rate $\nu_A = \nu_{m_A}\nu_{p_A}/\lambda_m$. Thus, each gene product in our models can be described by a single effective equation.

[1] Note that we consider protein degradation to occur at the same rate for monomers and dimers (no cooperative stability), such that the total concentration of a gene product degrades with the same rate as monomers and dimers, see e.g. Ref. [3].

2 Derivation of a reduced model for the genetic J-K latch with mutually exclusive heterodimer binding sites

Making the same quasi-equilibrium assumptions for protein-protein and protein-DNA as in Section I, the reduced model of the genetic J-K latch with mutually exclusive binding sites for the heterodimers KA and JB reads

$$\begin{aligned} \frac{dA_{\rm tot}}{dt} &= \alpha_A O_B(B_2)\, O_{KA}(KA, JB) - \lambda A_{tot}\,, \\ \frac{dB_{\rm tot}}{dt} &= \alpha_B O_A(A_2)\, O_{JB}(KA, JB) - \lambda B_{tot}\,, \end{aligned} \tag{8}$$

where only the operator occupancy functions of the heterodimers now also depend on the respective other heterodimer:

$$O_B(B_2) = \left(1 + \frac{B_2}{K_{B_2}}\right)^{-2}, \quad O_A(A_2) = \left(1 + \frac{A_2}{K_{A_2}}\right)^{-2}, \tag{9a}$$

$$O_{KA}(KA, JB) = \left(1 + \frac{KA}{K_{KA}(1 + (JB/K_{JB}))}\right)^{-1}, \tag{9b}$$

$$O_{JB}(KA, JB) = \left(1 + \frac{JB}{K_{JB}(1 + (KA/K_{KA}))}\right)^{-1}. \tag{9c}$$

However, in order to introduce a delay required for a successful toggle operation, the unbinding kinetics of the overlapping operator sites needs to be slow (see main text), such that the quasi-equilibrium assumption that lead to Eqs. (9a) and (9b) is not strictly valid. To describe the slow dynamics of the overlapping operator complex we set up the master equation for the occupancy states of the overlapping operators. There are three states to be accounted for: (i) the binding site for KA is occupied, (ii) the binding site for JB is occupied and (iii) both binding sites are unoccupied. We denote the probabilities of these states as q_{KA}, q_{JB} and q_0, respectively. The probabilities equivalent to Eqs. (9b) and (9c) to find the operator in an unoccupied state are then given by $O_{KA} = 1 - q_{KA}$ and $O_{JB} = 1 - q_{JB}$. Taking $k_{\rm on}$

to be the on rate of both operators and $k_{\rm off}$ the respective off-rate, the master equations read:

$$\dot{q}_{KA} = k_{\rm on} KA\; q_0 - k_{\rm off}\; q_{KA} \tag{10a}$$

$$\dot{q}_{JB} = k_{\rm on} JB\; q_0 - k_{\rm off}\; q_{JB} \tag{10b}$$

$$\dot{q}_0 = k_{\rm off}(q_{KA} + q_{JB}) - k_{\rm on}(KA + JB)\; q_0 \,. \tag{10c}$$

These equations are controlled by the external variables KA and JB. For given values of these variables, the system has a unique fixed point, which we denote by $(q^*_{KA}, q^*_{JB}, q^*_0)$. In the fixed point, the right hand side of Eq. (10) becomes zero, thus we can rewrite Eqs. (10a) and (10b) as

$$\dot{q}_{KA} = k_{\rm on} KA(q_0 - q^*_0) - k_{\rm off}(q_{KA} - q^*_{KA}) \tag{11a}$$

$$\dot{q}_{JB} = k_{\rm on} JB(q_0 - q^*_0) - k_{\rm off}(q_{JB} - q^*_{JB}) \,. \tag{11b}$$

Furthermore, we want to investigate Eqs. (10) under a constant toggle signal. Therefore, it is reasonable to assume that both heterodimers KA or JB are abundant in the system at all times. Then, the effective on-rates $k_{\rm on}KA$ and $k_{\rm on}JB$ are much faster than the off-rate $k_{\rm off}$ and thus the probability q_0 to find the overlapping operator sites unoccupied is approximately zero. Additionally, substituting $q_{KA} = 1 - O_{KA}$ and $q_{JB} = 1 - O_{JB}$, the approximated equations can be written as

$$\dot{O}_{KA} \approx -k_{\rm off}(O_{KA} - O^*_{KA}) \,, \tag{12a}$$

$$\dot{O}_{JB} \approx -k_{\rm off}(O_{JB} - O^*_{JB}) \,. \tag{12b}$$

We can identify the fixed points O^*_{KA} and O^*_{JB} with the quasi-equilibrium operator occupancy functions defined in Eqs. (9b) and (9c), which are functions of the time dependent heterodimer concentration. These equations can then be solved by variation of the constant:

$$O_{KA}(t) = \int_0^\infty k_{\rm off} e^{-k_{\rm off}(t-\tau)} O^*_{KA}(\tau)\, d\tau = \int_0^\infty g_k(\tau) O^*_{KA}(t-\tau)\, d\tau \,, \tag{13a}$$

$$O_{JB}(t) = \int_0^\infty k_{\rm off} e^{-k_{\rm off}(t-\tau)} O^*_{JB}(\tau)\, d\tau = \int_0^\infty g_k(\tau) O^*_{JB}(t-\tau)\, d\tau \,, \tag{13b}$$

with “memory kernel” $g_k(\tau) = k_{\rm off} e^{-k_{\rm off}\tau}$. Instead of accounting for the initial conditions in an own term,

we formally assume that for all negative times the system is held fixed at the initial point and integrate infinitely long into the past. This auxiliary assumption also simplifies the initial condition problem for the delayed dynamical system: for a distributed delay, instead of a single initial value, strictly the entire history prior to $t = 0$ has to be defined. Since we are interested in the qualitative long term behavior of the solution, we assume this history to be a single point from which the system is released at $t = 0$. Putting this result back into Eq. (8) leads to the delayed differential equations with exponentially distributed delay introduced in Fig. 3 Model 2.

Additional delays in the reduced model. For the following stability analysis, we include the additional delay caused by the translation process. While this is done for the sake of completeness, this additional delay is about ten times smaller than the delay caused by the overlapping heterodimer operators and is therefore not considered in the discussion of the main text. The additional delay arises from the reaction chain between the beginning of mRNA transcription and completion of protein translation. In principle, these kinds of delay alone can already lead to oscillations [4, 5]. Consider the rate equation for an arbitrary protein concentration A, with regulated transcription, linear degradation and an explicit equation for its mRNA denoted by m_A:

$$\frac{dA}{dt} = \nu_p m_A - \lambda_p A\,, \tag{14}$$

$$\frac{d\,m_A}{dt} = \nu_m P_A(t) - \lambda_m m_A\,. \tag{15}$$

Here, $P_A(t)$ denotes an arbitrary promoter activity function of gene A. Solving Eq. (15) and treating the initial conditions as above leads to

$$m_A(t) = \frac{\nu_m}{\lambda_m} \int_0^\infty \lambda_m e^{-\lambda_m \tau} P_A(t-\tau)\, d\tau\,. \tag{16}$$

Putting this back into Eq. 14 leads to

$$\frac{dA}{dt} = \nu_A \int_0^\infty g_\lambda(\tau) P_A(t-\tau)\, d\tau - \lambda_p A \tag{17}$$

with an effective expression rate $\nu_A = \nu_p \nu_m / \lambda_m$ and a delay kernel $g_\lambda(\tau) = \lambda_m e^{-\lambda_m \tau}$. Thus, taking the dynamics of mRNA into account, leads to an additional distributed delay acting on the entire promoter activity function. In a reduced model the protein concentration therefore responds to a change in promoter

activity on a timescale given by the mRNA degradation rate. This is generally valid for any gene controlled by regulated recruitment. There are, however, mechanisms of gene regulation, involving active degradation of mRNA [6, 7]. In that case, the dynamics of mRNA have to be accounted for by its full dynamic description.

3 Linear stability analysis on a system with distributed delays

Here we discuss linear stability analysis for nonlinear systems with multiple cascaded exponentially distributed delays, which closely follows the book of [8]. The results presented here are generally applicable to any system of that kind. The model system considered here is the simple J-K latch with overlapping heterodimer operators in Eqs. (8) and (9). As discussed in the last sections, it contains delays on the promoter activity functions P_A and P_B and, within those, on the operator occupation functions O_{KA} and O_{JB}. The memory kernels $g_k(\tau)$ and $g_\lambda(\tau)$ of these delays are exponential distributions with rate parameters k_{off} and λ_m, respectively.

To perform a linear stability analysis on our model system, it first is important to note that while delays change the stability of a fixed point, they do not alter their position with respect to the undelayed system. This is easy to see: consider a fixed point $FP = (A^*, B^*)$ of the undelayed system and the delayed function $\mathcal{D}_{g_k} \circ O_{KA}(A)$, where $\mathcal{D}_{g_k}$ abbreviates a distributed delay with memory kernel g. We assume that the delayed system has stayed in this fixed point for a very long time, such that the delayed function can be written as

$$\begin{aligned} \mathcal{D}_{g_k} \circ O_{KA}(A^*) &= \int_0^\infty g_k(\tau) O_{KA}\left(A^*(t-\tau)\right) \, d\tau \\ &= O_{KA}(A^*) \int_0^\infty g_k(\tau)\, d\tau = O_{KA}(A^*)\,, \end{aligned} \tag{18}$$

for any normalized kernel $g_k(\tau)$. Therefore, a fixed point of the undelayed system is a fixed point of the delayed system as well.

To perform a linear stability analysis on the model system Eq. (8), we must take into account that the dynamic variables A and B occur within different delay integrals and hence need to be treated separately in the linearization around a fixed point FP. Therefore, we categorize A and B by the delays acting on

them. The model system is

$$\dot{A} = \int_0^\infty g_\lambda(\tau) O_B^2(B_{g_\lambda}(t-\tau)) \left(\int_0^\infty g_k(\tau') O_{KA}(A_{g_{\lambda,k}}(t-\tau-\tau'), B_{g_{\lambda,k}}(t-\tau-\tau'))\, d\tau' \right) d\tau - \lambda_p A \tag{19}$$

$$\dot{B} = \int_0^\infty g_\lambda(\tau) O_A^2(A_{g_\lambda}(t-\tau)) \left(\int_0^\infty g_k(\tau') O_{JB}(A_{g_{\lambda,k}}(t-\tau-\tau'), B_{g_{\lambda,k}}(t-\tau-\tau'))\, d\tau' \right) d\tau - \lambda_p B \tag{20}$$

which can more formally be written as

$$\dot{A} = f\left(A, B_{g_\lambda}, A_{g_{\lambda,k}}, B_{g_{\lambda,k}}\right) , \tag{21}$$

$$\dot{B} = h\left(B, A_{g_\lambda}, A_{g_{\lambda,k}}, B_{g_{\lambda,k}}\right) . \tag{22}$$

Here, variables with subscript g_λ occur in functions, which are delayed only by mRNA degradation, whereas variables with subscript $g_{\lambda,k}$ are delayed by mRNA degradation and the off-rate of the overlapping operators. We now can linearize the system around a fixed point $FP = (A^*, B^*)$ and obtain linear equations for small deviations $\tilde{A} = A - A^*$ and $\tilde{B} = B - B^*$:

$$\frac{d\tilde{A}}{dt} = \left.\frac{\partial f}{\partial A}\right|_{FP} \tilde{A} + \mathcal{D}_{g_\lambda} \circ \left(\left.\frac{\partial f}{\partial B_{g_\lambda}}\right|_{FP} \tilde{B} + \mathcal{D}_{g_k} \circ \left(\left.\frac{\partial f}{\partial A_{g_{\lambda,k}}}\right|_{FP} \tilde{A} + \left.\frac{\partial f}{\partial B_{g_{\lambda,k}}}\right|_{FP} \tilde{B} \right) \right) , \tag{23}$$

$$\frac{d\tilde{B}}{dt} = \left.\frac{\partial h}{\partial B}\right|_{FP} \tilde{B} + \mathcal{D}_{g_\lambda} \circ \left(\left.\frac{\partial h}{\partial A_{g_\lambda}}\right|_{FP} \tilde{A} + \mathcal{D}_{g_k} \circ \left(\left.\frac{\partial h}{\partial B_{g_{\lambda,k}}}\right|_{FP} \tilde{B} + \left.\frac{\partial h}{\partial A_{g_{\lambda,k}}}\right|_{FP} \tilde{A} \right) \right) , \tag{24}$$

where $\mathcal{D}_{g_\lambda}$ denotes a delay with kernel $\lambda_m e^{-\lambda_m \tau}$ and $\mathcal{D}_{g_k}$ a delay with kernel $k_{\text{off}} e^{-k_{\text{off}}\tau}$. Since the position of the fixed point does not change for delayed variables, all partial derivatives are evaluated in

the same point FP. Taking into account that the functionals $\mathcal{D}$ are linear, the system can be written as

$$\begin{aligned}
\frac{d\tilde{A}}{dt} &= \left.\frac{\partial f}{\partial A}\right|_{FP} \tilde{A} \\
&+ \left.\frac{\partial f}{\partial B_{g_\lambda}}\right|_{FP} \mathcal{D}_{g_\lambda} \circ \tilde{B} + \left.\frac{\partial f}{\partial A_{g_{\lambda,k}}}\right|_{FP} \mathcal{D}_{g_\lambda} \circ \mathcal{D}_{g_k} \circ \tilde{A} + \left.\frac{\partial f}{\partial B_{g_{\lambda,k}}}\right|_{FP} \mathcal{D}_{g_\lambda} \circ \mathcal{D}_{g_k} \circ \tilde{B}\,, \qquad (25)\\
\frac{d\tilde{B}}{dt} &= \left.\frac{\partial h}{\partial B}\right|_{FP} \tilde{B} \\
&+ \left.\frac{\partial h}{\partial A_{g_\lambda}}\right|_{FP} \mathcal{D}_{g_\lambda} \circ \tilde{A} + \left.\frac{\partial h}{\partial B_{g_{\lambda,k}}}\right|_{FP} \mathcal{D}_{g_\lambda} \circ \mathcal{D}_{g_k} \circ \tilde{B} + \left.\frac{\partial h}{\partial A_{g_{\lambda,k}}}\right|_{FP} \mathcal{D}_{g_\lambda} \circ \mathcal{D}_{g_k} \circ \tilde{A}\,. \qquad (26)
\end{aligned}$$

Herein, the delayed variables take the form

$$\begin{aligned}
\mathcal{D}_{g_\lambda} \circ \tilde{A} &= \int_0^\infty \lambda e^{-\lambda\tau} \tilde{A}(t-\tau)\, d\tau\,, \qquad (27)\\
\mathcal{D}_{g_\lambda} \circ \mathcal{D}_{g_k} \circ \tilde{A} &= \int_0^\infty \lambda_m e^{-\lambda_m \tau} \left(\int_0^\infty k_{\text{off}} e^{-k_{\text{off}}\tau'} \tilde{A}(t-\tau-\tau')\, d\tau' \right) d\tau\,. \qquad (28)
\end{aligned}$$

We make the usual ansatz for a linear system: $\tilde{A} = c_A e^{zt}$ and $\tilde{B} = c_B e^{zt}$ with $z \in \mathbb{C}$ to be determined. Upon this ansatz, the delayed variables become

$$\mathcal{D}_{g_\lambda} \circ \tilde{A} = c_A \int_0^\infty \lambda_m e^{-\lambda_m \tau} e^{z(t-\tau)} = c_A e^{zt} \int_0^\infty \lambda_m e^{-\lambda_m \tau} e^{z\tau} = \tilde{A} \times \mathcal{L}(g_\lambda(\tau); z)\,, \qquad (29)$$

where $\mathcal{L}(g_\lambda(\tau); z)$ denotes the Laplace transform of the delay kernel. This holds for any normalized delay kernel. Since $g_\lambda(\tau)$ is an exponential distribition, its Laplace transform takes a particularly simple form:

$$\mathcal{L}(g_\lambda(\tau); z) = \frac{\lambda_m}{\lambda_m + z} \qquad \Rightarrow \qquad \mathcal{D}_{g_\lambda} \circ \tilde{A} = \tilde{A} \frac{\lambda_m}{\lambda_m + z}\,. \qquad (30)$$

Variables with two delays can be evaluated in a similar way:

$$\begin{aligned}
\mathcal{D}_{g_\lambda} \circ \mathcal{D}_{g_k} \circ \tilde{A} &= c_A \int_0^\infty \lambda_m e^{-\lambda_m \tau} \left(\int_0^\infty k_{\text{off}} e^{-k_{\text{off}}\tau'} e^{z(t-\tau-\tau')}\, d\tau' \right) d\tau \\
&= \tilde{A} \int_0^\infty \lambda_m e^{-\lambda_m \tau} e^{-z\tau}\, d\tau \times \int_0^\infty k_{\text{off}} e^{-k_{\text{off}}\tau'} e^{-z\tau}\, d\tau' \\
&= \tilde{A}\, \frac{\lambda_m}{\lambda_m + z}\, \frac{k_{\text{off}}}{k_{\text{off}} + z}\,. \qquad (31)
\end{aligned}$$

Putting these results back into Eqs. 25 and 26 yields a linear equation for the coefficients c_A and c_B:

$$\begin{pmatrix} c_A z \\ c_B z \end{pmatrix} = \begin{pmatrix} \frac{\partial f}{\partial A} + \frac{\partial f}{\partial A_{g_{\lambda,k}}} \frac{\lambda_m}{\lambda_m + z} \frac{k_{\text{off}}}{k_{\text{off}} + z} & \frac{\partial f}{\partial B_1^D} \frac{\lambda_m}{\lambda_m + z} + \frac{\partial f}{\partial B_{g_{\lambda,k}}} \frac{\lambda_m}{\lambda_m + z} \frac{k_{\text{off}}}{k_{\text{off}} + z} \\ \frac{\partial h}{\partial A_{g_\lambda}} \frac{\lambda_m}{\lambda_m + z} + \frac{\partial h}{\partial B_{g_{\lambda,k}}} \frac{\lambda_m}{\lambda_m + z} \frac{k_{\text{off}}}{k_{\text{off}} + z} & \frac{\partial h}{\partial B} + \frac{\partial h}{\partial B_{g_{\lambda,k}}} \frac{\lambda_m}{\lambda_m + z} \frac{k_{\text{off}}}{k_{\text{off}} + z} \end{pmatrix} \begin{pmatrix} c_A \\ c_B \end{pmatrix}. \tag{32}$$

Note that all partial derivatives have to be evaluated at the fixed point under consideration. Subtracting the left hand side from the diagonal elements of the right hand side leads to the familiar form a an eigenvalue problem. Here, in contrast to a common linear stability analysis, additional polynomial terms are incorporated, representing the delays. In order for the system to have a solution, its determinant has to be zero. Thereby we obtain a characteristic equation for the exponents z.

$$\left| \begin{pmatrix} \frac{\partial f}{\partial A} + \frac{\partial f}{\partial A_{g_{\lambda,k}}} \frac{\lambda_m}{\lambda_m + z} \frac{k_{\text{off}}}{k_{\text{off}} + z} - z & \frac{\partial f}{\partial B_1^D} \frac{\lambda_m}{\lambda_m + z} + \frac{\partial f}{\partial B_{g_{\lambda,k}}} \frac{\lambda_m}{\lambda_m + z} \frac{k_{\text{off}}}{k_{\text{off}} + z} \\ \frac{\partial h}{\partial A_{g_\lambda}} \frac{\lambda_m}{\lambda_m + z} + \frac{\partial h}{\partial B_{g_{\lambda,k}}} \frac{\lambda_m}{\lambda_m + z} \frac{k_{\text{off}}}{k_{\text{off}} + z} & \frac{\partial h}{\partial B} + \frac{\partial h}{\partial B_{g_{\lambda,k}}} \frac{\lambda_m}{\lambda_m + z} \frac{k_{\text{off}}}{k_{\text{off}} + z} - z \end{pmatrix} \right| = 0\,. \tag{33}$$

Evaluating the determinant, we obtain a polynomial of 6th order in z. In order for the considered fixed point to be stable, each solution of Eq. 33 must have a negative real part. The timescales of the system considered here are given by $\overline{\tau}_\lambda = 1/\lambda_m$ and $\overline{\tau}_k = 1/k_{\text{off}}$, which are the mean values of the memory kernel. These characteristic times are usually referred to as the mean delay [8]. Written in terms of the mean delay $\overline{\tau}$, the Laplace transform of an exponential distribution becomes $1/(1 + \overline{\tau} z)$. Thus, if the delay is small, its contributing terms in the characteristic equation are close to one and therefore do not change the stability of a fixed point. In the following we will employ the derived method of linear stability analysis to investigate the genetic JK-latch with overlapping heterodimer operators.

4 Analysis of deterministic and stochastic oscillations in the genetic JK-latch

To analyze the stability properties of the genetic JK-latch with overlapping heterodimer operators for a certain choice of parameters and concentration of input proteins J_{tot} and K_{tot}, we applied the following procedure using Mathematica (Wolfram Research, Inc.): (i) calculate the concentrations of all homo- and heterodimers using Eqs. (2)-(4). For the stability analysis we manipulate the concentration of input proteins J_{tot} and K_{tot} directly, instead of controlling their transcription rate. (ii) Find all fixed points by

setting Eq. (8) to zero. (iii) For each fixed point solve the characteristic equation defined in Eq. (31). If the real parts of all solutions of the last step are negative, the fixed point is stable otherwise it is unstable. (iv) If necessary, characterize the system's qualitative behavior (e.g. oscillatory) by solving its equations numerically. Employing this scheme for different parameter values we identify two important bifurcations: the system undergoes a Hopf bifurcation [9, 10] if the concentration of input proteins J_{tot} and K_{tot} is increased (see Fig. S2), then is oscillatory for a certain concentration range until it becomes stable again. Additionally, the system exhibits another Hopf bifurcation if the mean delay time is increased (i.e. the parameter k_{off} is decreased), while a constant toggle signal is applied. This is illustrated in Fig. S3A: at a critical operator dwell time $\tau_{crit} \approx 32$ min the system becomes oscillatory with a period that increases with the dwell time.

In the form we set up the reduced model for the genetic JK-latch with overlapping heterodimer operators, we couple the dynamics of protein concentrations with the intrinsically fluctuating switching of operator states. Therefore, although the deterministic model provides valuable insights into the working principles of the circuit, the quantitative period of the stochastic system is not correctly reproduced. This is shown in Fig. 3B, where it is apparent that the average of the stochastic period is much shorter than the period of the deterministic model. Also the stochastic system displays oscillations in a regime of short heterodimer dwell times, in which the deterministic system does not oscillate at all. In fact, it is known that a small number of reactant molecules together with negative feedback and time delay in gene expression can lead to delay-induced instabilities, such that a system turns oscillatory even when its deterministic counterpart is not [4].

If the delay (dwell time) is long, the strength of the toggle signal is chosen appropriately and the signal duration is well timed, then high probabilities of a correct toggle response can be achieved – as can be seen in Fig. S4A and B.

5 Genetic master-slave latch

We extended the reaction system of the JK-latch to a master-slave latch by adding additional regulation of the signal genes J and K – see Table S4. This was done by firstly including homodimerization of signal proteins. J_2 and K_2 bind two operators on the promoter of the respective other gene to repress it. Additionally, gene J is repressed by heterodimers KA by binding to a third operator in its promoter

region, while gene K is repressed by JB in the same way. Notably, the restrictive promoter layout of the overlapping heterodimer operators on genes A and B with very slow unbinding rates is no longer necessary in the master-slave latch and is changed to independent operators with normal unbinding rates.

With this additional regulation, signal genes J and K become bistable when their maximal transcription rate is increased externally (by the toggle signal) and thereby form the master toggle switch. Then, the master toggle switch settles into a state that is determined by the current concentrations of heterodimers and therefore by the current state of the slave latch. Dependent on the operator strengths for homodimers and for heterodimers two kinds of erroneous responses are possible upon a toggle signal: If homodimers bind to strongly with respect to heterodimers, then the master switch is prone to stochastic fluctuations at the onset of the toggle signal and biased towards that signal protein which is first abundant enough to form homodimers. If, on the other hand, heterodimer binding is too strong, random single bursts of the repressed signal gene can be sufficient to switch the state of the master latch. Fig. S4C shows the toggle probability as a function of those key parameters for a toggle signal duration of 60 min. It can be seen that the master-slave latch is robust in that sense, that it responds correctly for a broad range of those parameters.

**JK-latch with overlapping heterodimer operators**	
Promoter and operator states	
$P_A\|\cdot\|\cdot + B_2 \xrightarrow{k^{\mathrm{on}}_{O_{B2}}} P_A\|B_2\|\cdot$	$P_A\|B_2\|\cdot \xrightarrow{k^{\mathrm{off}}_{O_{B2}}} P_A\|\cdot\|\cdot + B_2$
$P_A\|\cdot\|\cdot + B_2 \xrightarrow{k^{\mathrm{on}}_{O_{B2}}} P_A\|\cdot\|B_2$	$P_A\|\cdot\|B_2 \xrightarrow{k^{\mathrm{off}}_{O_{B2}}} P_A\|\cdot\|\cdot + B_2$
$P_A\|\cdot\|B_2 + B_2 \xrightarrow{k^{\mathrm{on}}_{O_{B2}}} P_A\|B_2\|B_2$	$P_A\|B_2\|B_2 \xrightarrow{k^{\mathrm{off}}_{O_{B2}}} P_A\|\cdot\|B_2 + B_2$
$P_A\|B_2\|\cdot + B_2 \xrightarrow{k^{\mathrm{on}}_{O_{B2}}} P_A\|B_2\|B_2$	$P_A\|B_2\|B_2 \xrightarrow{k^{\mathrm{off}}_{O_{B2}}} P_A\|B_2\|\cdot + B_2$
$P_B\|\cdot\|\cdot + A_2 \xrightarrow{k^{\mathrm{on}}_{O_{A2}}} P_B\|A_2\|\cdot$	$P_B\|A_2\|\cdot \xrightarrow{k^{\mathrm{off}}_{O_{A2}}} P_B\|\cdot\|\cdot + A_2$
$P_B\|\cdot\|\cdot + A_2 \xrightarrow{k^{\mathrm{on}}_{O_{A2}}} P_B\|\cdot\|A_2$	$P_B\|\cdot\|A_2 \xrightarrow{k^{\mathrm{off}}_{O_{A2}}} P_B\|\cdot\|\cdot + A_2$
$P_B\|\cdot\|A_2 + A_2 \xrightarrow{k^{\mathrm{on}}_{O_{A2}}} P_B\|A_2\|A_2$	$P_B\|A_2\|A_2 \xrightarrow{k^{\mathrm{off}}_{O_{A2}}} P_B\|\cdot\|A_2 + A_2$
$P_B\|A_2\|\cdot + A_2 \xrightarrow{k^{\mathrm{on}}_{O_{A2}}} P_B\|A_2\|A_2$	$P_B\|A_2\|A_2 \xrightarrow{k^{\mathrm{off}}_{O_{A2}}} P_B\|A_2\|\cdot + A_2$
$O\|\cdot\|\cdot + KA \xrightarrow{k^{\mathrm{on}}_{O_{KA}}} O\|KA\|\cdot$	$O\|KA\|\cdot \xrightarrow{k^{\mathrm{off}}_{O_{KA}}} O\|\cdot\|\cdot + KA$
$O\|\cdot\|\cdot + JB \xrightarrow{k^{\mathrm{on}}_{O_{KA}}} O\|\cdot\|JB$	$O\|\cdot\|JB \xrightarrow{k^{\mathrm{off}}_{O_{KA}}} O\|\cdot\|\cdot + JB$

Dimerization	
$2A \xrightarrow{k_A^{\mathrm{on}}} A_2$	$J + A \xrightarrow{k_J^{\mathrm{on}}} KA$
$2B \xrightarrow{k_B^{\mathrm{on}}} B_2$	$K + B \xrightarrow{k_K^{\mathrm{on}}} JB$
$A_2 \xrightarrow{k_A^{\mathrm{off}}} 2A$	$KA \xrightarrow{k_J^{\mathrm{off}}} J + A$
$B_2 \xrightarrow{k_B^{\mathrm{off}}} 2B$	$JB \xrightarrow{k_K^{\mathrm{off}}} K + B$
Turnover	
$m_A \xrightarrow{\lambda_{m_A}} \emptyset$	$A_2 \xrightarrow{\lambda_p} \emptyset$
$m_B \xrightarrow{\lambda_{m_B}} \emptyset$	$B_2 \xrightarrow{\lambda_p} \emptyset$
$m_J \xrightarrow{\lambda_m} \emptyset$	$J \xrightarrow{\lambda_p} \emptyset$
$m_K \xrightarrow{\lambda_m} \emptyset$	$K \xrightarrow{\lambda_p} \emptyset$
$A \xrightarrow{\lambda_p} \emptyset$	$KA \xrightarrow{\lambda_p} \emptyset$
$B \xrightarrow{\lambda_p} \emptyset$	$JB \xrightarrow{\lambda_p} \emptyset$
Transcription and translation	
$P_J \xrightarrow{\nu_{m_J}} P_J + m_J$	$m_A \xrightarrow{\nu_{p_A}} m_A + A$
$P_K \xrightarrow{\nu_{m_K}} P_K + m_K$	$m_B \xrightarrow{\nu_{p_B}} m_B + B$
$P_A\vert\cdot\vert\cdot + O\vert\cdot\vert\cdot \xrightarrow{\nu_{m_A}} P_A\vert\cdot\vert\cdot + O\vert\cdot\vert\cdot + m_A$	$m_J \xrightarrow{\nu_{p_J}} m_J + J$
$P_B\vert\cdot\vert\cdot + O\vert\cdot\vert\cdot \xrightarrow{\nu_{m_B}} P_B\vert\cdot\vert\cdot + O\vert\cdot\vert\cdot + m_B$	$m_K \xrightarrow{\nu_{p_K}} m_K + K$
$P_A\vert\cdot\vert\cdot + O\vert\cdot\vert JB \xrightarrow{\nu_{m_A}} P_A\vert\cdot\vert\cdot + O\vert\cdot\vert JB + m_A$	
$P_B\vert\cdot\vert\cdot + O\vert KA\vert\cdot \xrightarrow{\nu_{m_B}} P_B\vert\cdot\vert\cdot + O\vert KA\vert\cdot + m_B$	

Table S1. All reactions involved in the genetic JK-latch with overlapping heterodimer binding sites. A graphical representation of the reaction network is shown in Fig. S1. Proteins and their dimers are denoted by capital letters; transcripts of a gene X are denoted by m_X. A gene X is represented by its promoter P_X, which can be occupied by transcriptions factors. Each occupation state of a promoter is represented by an own species of reactants for which an empty operator is indicated by $\cdot$ and an occupied operator by the name of the respective transcription factor. In this notation the different binding sites are separated by the symbol $\vert$. To reduce the number of occupation state combinations the operator complex for heterodimers is separated from the other promoter states and denoted by O. To make transcription nevertheless conditional on heterodimers, we include the respective species for an empty binding site as reactant and product into the corresponding transcription reaction. This ensures that transcription requires an empty heterodimer operator to proceed but does not change its concentration. All corresponding parameter values are listed in Table S3.

**Additional reactions in J-K latch without overlapping heterodimer operators**	
$O\vert\cdot\vert JB + KA \xrightarrow{k_{O_{KA}}^{\mathrm{on}}} O\vert KA\vert JB$	$O\vert KA\vert JB \xrightarrow{k_{O_{KA}}^{\mathrm{off}}} O\vert\cdot\vert JB + KA$
$O\vert KA\vert\cdot + JB \xrightarrow{k_{O_{JB}}^{\mathrm{on}}} O\vert KA\vert JB$	$O\vert KA\vert JB \xrightarrow{k_{O_{JB}}^{\mathrm{off}}} O\vert KA\vert\cdot + JB$

Table S2. With independent heterodimer operators, heterodimers can bind simultaneously to their binding sites. All other reactions are the same as in the JK-latch with overlapping heterodimer operators, listed in Table S1.

Parameter	Value	Description and References
**Transcription**		
ν_{m_A}, ν_{m_B}	$5\ min^{-1}$	strong promoter [11]
ν_{m_J}, ν_{m_K}	$(0.01-1)\ min^{-1}$	inducible promoter [11]; induction can be achieved e.g. by upstream binding activators [12] or via small non-coding RNAs [13]
**Translation**		
ν_{p_A}, ν_{p_B}, ν_{p_J}, ν_{p_K}	$0.23\ min^{-1}$	corresponds to a burst factor of $b=10$ [14]
**Degradation**		
λ_m	$0.23\ min^{-1}$	active degradation by RNase [15]; half-life of 3 min
λ_p	$0.138\ min^{-1}$	active proteolysis by SsrA-tags [16]; half life of 5 min
**Dimerization**		
k_A^{on}, k_B^{on}, k_{KA}^{on}, k_{JB}^{on}	$0.2\ nM^{-1}min^{-1}$	assumed to be diffusion limited [17]
k_A^{off}, k_B^{off}, k_J^{off}, k_K^{off}	$2\ min^{-1}$	typical value for dimerization
**Protein-DNA binding**		
$k_{O_{A2}}^{\text{on}}$, $k_{O_{B2}}^{\text{on}}$	$0.2\ nM^{-1}min_{-1}$	assumed to be diffusion limited [17]
$k_{O_{A2}}^{\text{off}}$, $k_{O_{B2}}^{\text{off}}$	$2\ min^{-1}$	modified operator sequence assumed; parameter in the range of tet-O_1 binding strength [18]
$k_{O_{KA}}^{\text{on}}$, $k_{O_{JB}}^{\text{on}}$	$0.2\ nM^{-1}min^{-1}$	assumed to be diffusion limited [17]
$k_{O_{KA}}^{\text{off}}$, $k_{O_{JB}}^{\text{off}}$	$0.0126\ min^{-1}$	very slow off-rate assumed [19, 20]

Table S3. Values and references for all parameters used in the reactions of the genetic JK-latch.

Additional reactions in master-slave latch	
Promoter and operator states	
$P_J\|\cdot\|\cdot + K_2 \xrightarrow{s^{\text{on}}_{O_{K2}}} P_J\|K_2\|\cdot$	$P_J\|K_2\|\cdot \xrightarrow{s^{\text{off}}_{O_{K2}}} P_J\|\cdot\|\cdot + K_2$
$P_J\|\cdot\|\cdot + K_2 \xrightarrow{s^{\text{on}}_{O_{K2}}} P_J\|\cdot\|K_2$	$P_J\|\cdot\|K_2 \xrightarrow{s^{\text{off}}_{O_{K2}}} P_J\|\cdot\|\cdot + K_2$
$P_J\|\cdot\|K_2 + K_2 \xrightarrow{s^{\text{on}}_{O_{K2}}} P_J\|K_2\|K_2$	$P_J\|K_2\|K_2 \xrightarrow{s^{\text{off}}_{O_{K2}}} P_J\|\cdot\|K_2 + K_2$
$P_J\|K_2\|\cdot + K_2 \xrightarrow{s^{\text{on}}_{O_{K2}}} P_J\|K_2\|K_2$	$P_J\|K_2\|K_2 \xrightarrow{s^{\text{off}}_{O_{K2}}} P_J\|K_2\|\cdot + K_2$
$P_K\|\cdot\|\cdot + J_2 \xrightarrow{s^{\text{on}}_{O_{J2}}} P_K\|J_2\|\cdot$	$P_K\|J_2\|\cdot \xrightarrow{s^{\text{off}}_{O_{J2}}} P_K\|\cdot\|\cdot + J_2$
$P_K\|\cdot\|\cdot + J_2 \xrightarrow{s^{\text{on}}_{O_{J2}}} P_K\|\cdot\|J_2$	$P_K\|\cdot\|J_2 \xrightarrow{s^{\text{off}}_{O_{J2}}} P_K\|\cdot\|\cdot + J_2$
$P_K\|\cdot\|J_2 + J_2 \xrightarrow{s^{\text{on}}_{O_{J2}}} P_K\|J_2\|J_2$	$P_K\|J_2\|J_2 \xrightarrow{s^{\text{off}}_{O_{J2}}} P_K\|\cdot\|J_2 + J_2$
$P_K\|J_2\|\cdot + J_2 \xrightarrow{s^{\text{on}}_{O_{J2}}} P_K\|J_2\|J_2$	$P_K\|J_2\|J_2 \xrightarrow{s^{\text{off}}_{O_{J2}}} P_K\|J_2\|\cdot + J_2$
$O_J\|\cdot + KA \xrightarrow{s^{\text{on}}_{O_{KA}}} O_J\|KA$	$O_J\|KA \xrightarrow{s^{\text{off}}_{O_{KA}}} O_J\|\cdot + KA$
$O_K\|\cdot + JB \xrightarrow{s^{\text{on}}_{O_{JB}}} O_K\|JB$	$O_K\|JB \xrightarrow{s^{\text{off}}_{O_{JB}}} O_K\|\cdot + JB$
Dimerization	
$2J \xrightarrow{s^{\text{on}}_J} J_2$	$J_2 \xrightarrow{s^{\text{off}}_J} 2J$
$2K \xrightarrow{s^{\text{on}}_K} K_2$	$K_2 \xrightarrow{s^{\text{off}}_K} 2K$
Turnover	
$J_2 \xrightarrow{\lambda_p} \emptyset$	$K_2 \xrightarrow{\lambda_p} \emptyset$
Transcription	
$P_J\|\cdot\|\cdot + O_J\|\cdot \xrightarrow{\nu_{m_J}} P_J\|\cdot\|\cdot + O_J\|\cdot + m_J$	$P_K\|\cdot\|\cdot + O_K\|\cdot \xrightarrow{\nu_{m_K}} P_K\|\cdot\|\cdot + O_K\|\cdot + m_K$

Table S4. Additional reactions involved in regulation of genes J and K. All other reactions are the same as in the genetic JK-latch with independent heterodimer operators. Values of new parameters are listed in Table S5.

Parameter	Value	Description and References
**Transcription**		
ν_{m_J}, ν_{m_K}	$(0.05-5)\ min^{-1}$	strong inducible promoter [11]; induction can be achieved e.g. by upstream binding activators [12] or via small non-coding RNAs [13]
**Dimerization**		
s_J^{on}, s_K^{on}	$0.2\ nM^{-1}min^{-1}$	assumed to be diffusion limited [17]
s_J^{off}, s_K^{off}	$2\ min^{-1}$	equal to dimerization of A and B
**Protein-DNA binding**		
$s_{O_{J2}}^{\text{on}}$, $s_{O_{K2}}^{\text{on}}$	$0.2\ nM^{-1}min_{-1}$	diffusion limited [17]
$s_{O_{J2}}^{\text{off}}$, $s_{O_{K2}}^{\text{off}}$	$2\ min^{-1}$	optimized for highest toggle probability
$s_{O_{KA}}^{\text{on}}$, $s_{O_{JB}}^{\text{on}}$	$0.2\ nM^{-1}min^{-1}$	binding to the operator on the promoter
$s_{O_{KA}}^{\text{off}}$, $s_{O_{JB}}^{\text{off}}$	$0.35\ min^{-1}$	optimized for highest toggle probability
**Changes in slave circuit**		
$k_{O_{KA}}^{\text{off}}$, $k_{O_{JB}}^{\text{off}}$	$2\ min^{-1}$	use same binding strength as homodimers

Table S5. Additional parameters used in the genetic master-slave latch. Instead of the symbol k for rates in the slave circuit, the symbol s is used for rates of all addtional processes (dimerization and protein-DNA binding of signal proteins) in the master circuit. Since a delay in heterodimer binding is no longer needed, the assumption of very slow unbinding kinetics of heterodimers from promoters of genes A and B has been released.

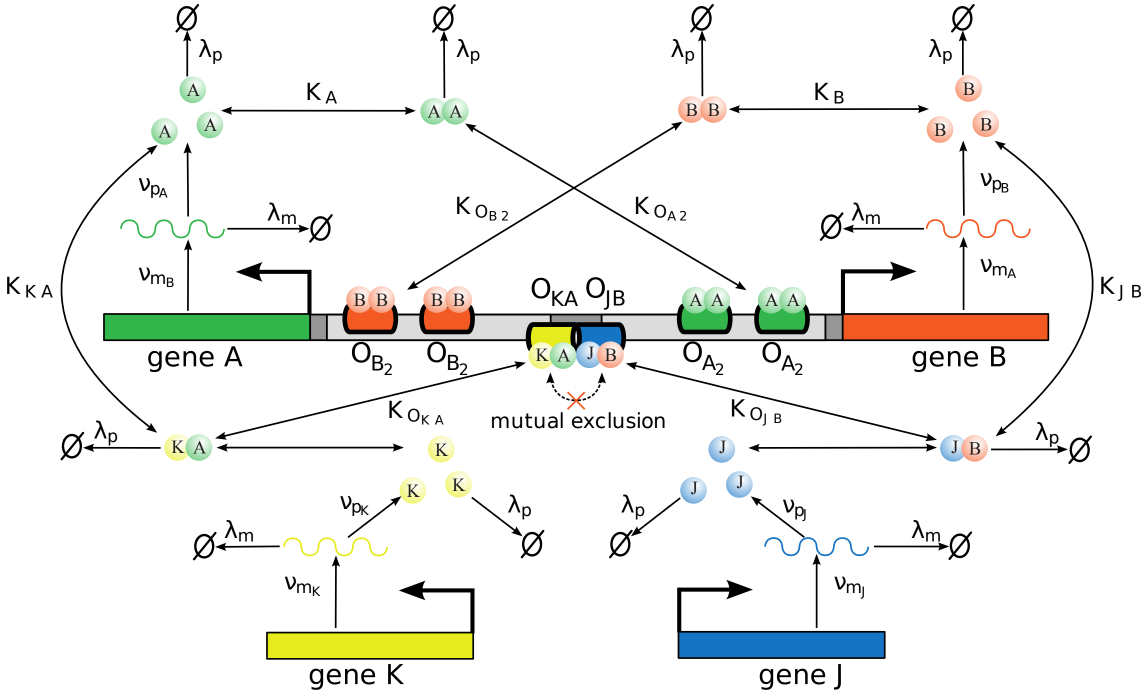

Figure S1. Schematic of the reaction network of the JK-latch with overlapping heterodimer operators. All reactions contained in the model are depicted together with their rates. On- and off-rates (k^{on} and k^{off}) are combined to dissociation constants $K = k^{\mathrm{off}}/k^{\mathrm{on}}$.

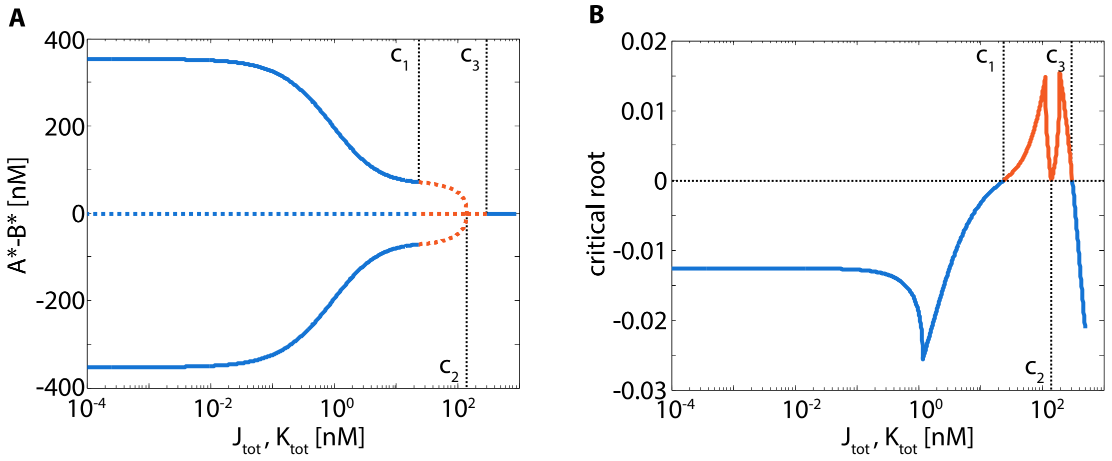

Figure S2. **(A)** Bifurcation diagram of the genetic JK-latch with overlapping heterodimer operators. The relative position of the system's fixed points is shown with the total concentrations of input proteins J_{tot} and K_{tot} as synchronously tuned bifurcation parameter. **(B)** Value of the critical root calculated in the linear stability analysis, presented in Section 3, for the upmost fixed point (ON state) in Fig. **(A)**. The root is critical in that sense that it is the only solution of the characteristic equation, which becomes positive for certain values of $J_{\mathrm{tot}}/K_{\mathrm{tot}}$. Starting from a low concentration of input proteins the system initially has two stable (solid line) and one unstable (dotted line) fixed point. The upper stable fixed point represents the ON state, whereas the lower one represents the OFF state. As the concentration of input proteins is increased, the two stable fixed points loose their stability at a critical concentration c_1, after which delay induced oscillations commence (orange lines). At input protein concentration c_2 the three fixed points collapse to one. That, however, does not alter the system's oscillatory behavior. At concentration c_3 the system's single fixed point becomes stable again. This is due to depletion of homodimers by increasingly forming heterodimers, which eventually abolishes the switch-like behavior of genes A and B.

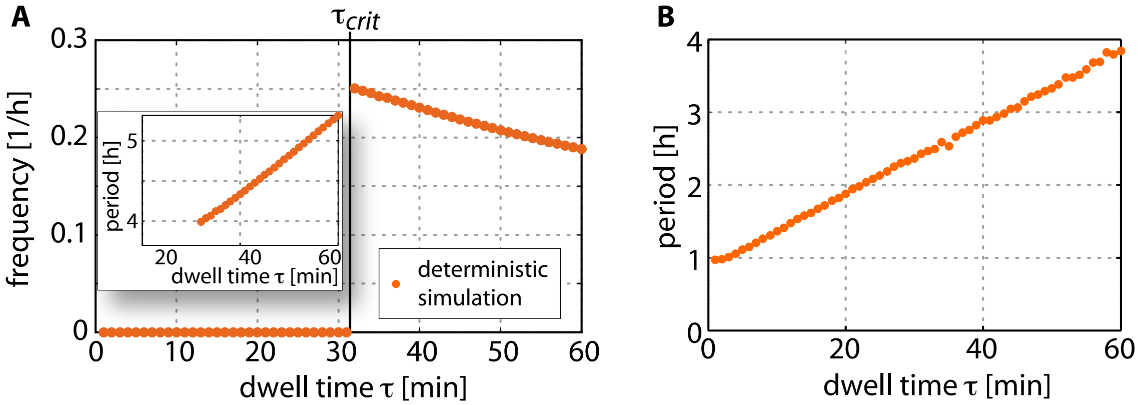

Figure S3. **(A)** Frequency of the race condition as a function of the average dwell time τ of a heterodimer on the overlapping operator complex (inset shows the period of the same). At a critical dwell time $\tau_{crit} \approx 32$ min the system undergoes a Hopf bifurcation and thereafter oscillates with a period that is approximately linear in τ. **(B)** Average period of the stochastic race condition as a function of the mean dwell time τ. In comparison to **(A)** the stochastic system oscillates even for small dwell times (although with very low amplitude) and has a shorter period than the deterministic model. The latter is due to the fact that the deterministic model couples the dynamics of protein concentrations with probabilities of operator switching, which has a distorting effect on the oscillatory dynamics.

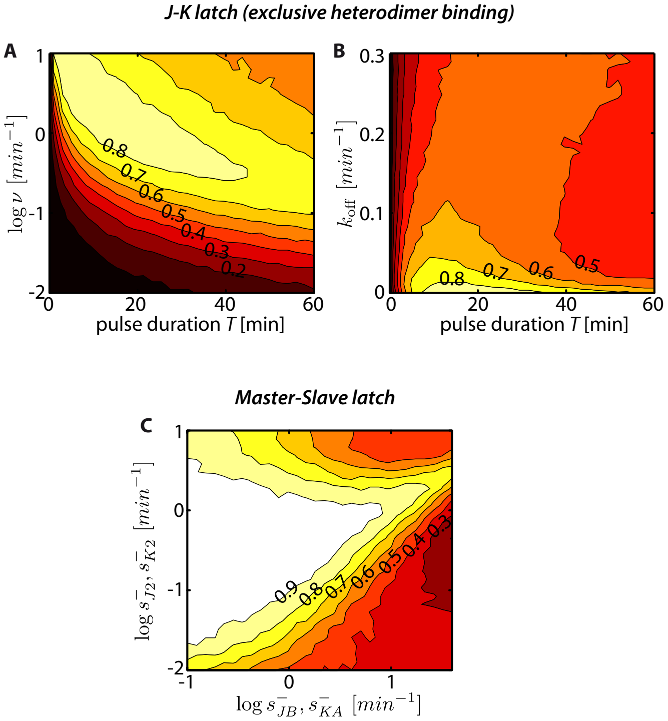

Figure S4. Toggle probability p_{toggle} as a function of key parameters. Contours indicate equal probability to toggle successfully into the complementary state. Each data point (a grid of 30 by 30 per plot) is estimated by testing the final state of 5000 simulation runs of the respective full stochastic model. **(A)** Dependence of p_{toggle} on the duration T and the strength of the toggle signal in the JK-latch. Here the strength of the toggle signal is tuned by a concerted variation of the transcription rates of genes J and K, i.e., $\nu_J = \nu_K = \nu$. **(B)** Dependence of p_{toggle} on the duration T of the toggle signal and the off-rate k_{off} for unbinding the overlapping operator sites. As expected, p_{toggle} increases as the delay (dwell time of heterodimers on the binding site) is increased. **(C)** Dependence of p_{toggle} in the master-slave latch on the additional rates s^{off}_{J2}, s^{off}_{K2} for unbinding the homodimer operators and s^{off}_{JB}, s^{off}_{KA} for unbinding the heterodimer operators in the additional toggle switch (master latch). The master-slave latch is robust in that sense that p_{toggle} is high for a broad range of parameters.

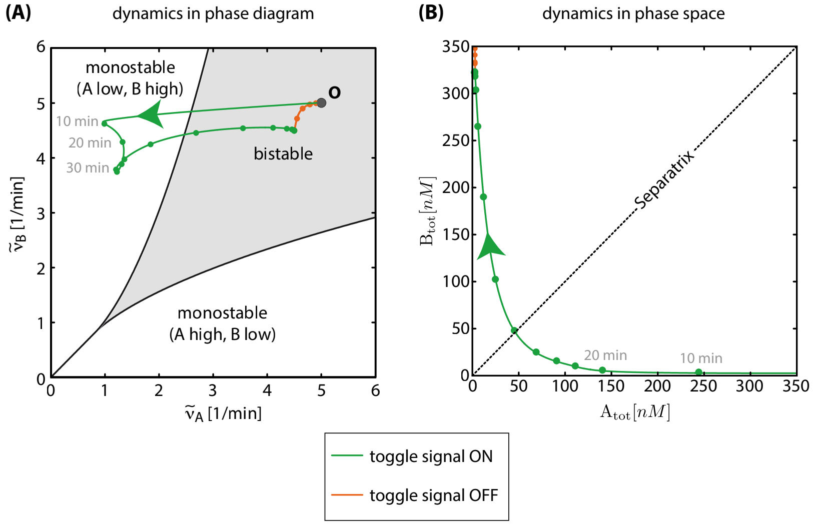

Figure S5

Dynamics of the deterministic model of the master-slave latch. **(A)** Phase diagram of the toggle switch as a function of the effective maximal transcription rates $\tilde{\nu}_{\mathrm{A}}$ and $\tilde{\nu}_{\mathrm{B}}$. The parameters are chosen such that the system is in the bistable regime in the absence of input signals (*point* **O**) and the circuit is set to the ON state initially. The curves indicate dynamic changes of $\tilde{\nu}_{\mathrm{A}}$ and $\tilde{\nu}_{\mathrm{B}}$, incurred by applying the toggle signal (simultaneous expression of both input genes J and K) for 200 min (*green solid curve*) and then releasing it (*red curve*). In contrast to the JK-latch, even if the toggle signal is applied a long time, the system enters the correct monostable regime (A low, B high), switches to the OFF state and returns to the bistable region without approaching the other monostable regime. In particular, the master-slave latch does not oscillate, even under a continuous toggle signal. **(B)** The same trajectories in the ([$\mathrm{A_{tot}}$],[$\mathrm{B_{tot}}$])-plane.

References

1. Bintu L, Buchler NE, Garcia HG, Gerland U, Hwa T, et al. (2005) Transcriptional regulation by the numbers: models. Curr Opin Genet Dev 15: 116–124.

2. Bintu L, Buchler NE, Garcia HG, Gerland U, Hwa T, et al. (2005) Transcriptional regulation by the numbers: applications. Curr Opin Genet Dev 15: 125–135.

3. Buchler NE, Gerland U, Hwa T (2005) Nonlinear protein degradation and the function of genetic circuits. Proc Natl Acad Sci U S A 102: 9559–9564.

4. Bratsun D, Volfson D, Tsimring LS, Hasty J (2005) Delay-induced stochastic oscillations in gene regulation. Proc Natl Acad Sci U S A 102: 14593–14598.

5. Lewis J (2003) Autoinhibition with transcriptional delay: A simple mechanism for the zebrafish somitogenesis oscillator. Curr Biol 13: 1398–1408.

6. Atwater JA, Wisdom R, Verma IM (1990) Regulated messenger-RNA stability. Annu Rev Genet 24: 519–541.

7. Sittman DB, Graves RA, Marzluff WF (1983) Histone messenger-RNA concentrations are regulated at the level of transcription and messenger-RNA degradation. Proc Natl Acad Sci U S A 80: 1849–1853.

8. MacDonald N (1989) Biological delay systems: linear stability theory. Cambridge University Press.

9. Strogatz SH (1994) Nonlinear dynamics and shaos: with applications to physics, biology, chemistry and engineering. Perseus Books Group.

10. Wiggins S (2003) Introduction to applied nonlinear dynamical systems and chaos. Springer.

11. Lutz R, Bujard H (1997) Independent and tight regulation of transcriptional units in *Escherichia coli* via the LacR/O, the TetR/O and AraC/I-1-I-2 regulatory elements. Nucleic Acids Res 25: 1203–1210.

12. Ptashne M, Gann A (1997) Transcriptional activation by recruitment. Nature 386: 569–577.

13. Storz G, Vogel J, Wassarman KM (2011) Regulation by small RNAs in bacteria: expanding frontiers. Mol Cell 43: 880–891.

14. Thattai M, van Oudenaarden A (2001) Intrinsic noise in gene regulatory networks. Proc Natl Acad Sci U S A 98: 8614–8619.

15. Bernstein JA, Khodursky AB, Lin PH, Lin-Chao S, Cohen SN (2002) Global analysis of mRNA decay and abundance in *Escherichia coli* at single-gene resolution using two-color fluorescent DNA microarrays. Proc Natl Acad Sci U S A 99: 9697–9702.

16. Gottesman S, Roche E, Zhou YN, Sauer RT (1998) The ClpXP and ClpAP proteases degrade proteins with carboxy-terminal peptide tails added by the SsrA-tagging system. Genes Dev 12: 1338–1347.

17. Bruinsma RF (2002) Physics of protein-DNA interaction. Physica A 313: 211–237.

18. Fritz G, Buchler NE, Hwa T, Gerland U (2007) Designing sequential transcription logic: a simple genetic circuit for conditional memory. Syst Synth Biol 1: 89–98.

19. Vershon AK, Liao SM, McClure WR, Sauer RT (1987) Bacteriophage P22 Mnt repressor DNA-binding and effects on transcription *in vitro*. J Mol Biol 195: 311–322.

20. Dong F, Spott S, Zimmermann O, Kisters-Woike B, Müller-Hill BM, et al. (1999) Dimerisation mutants of lac repressor. I. a monomeric mutant, L251A, that binds lac operator DNA as a dimer. J Mol Biol 290: 653–666.
